# Natriuretic peptides for the detection of diastolic dysfunction and heart failure with preserved ejection fraction—a systematic review and meta-analysis

**DOI:** 10.1186/s12916-020-01764-x

**Published:** 2020-10-30

**Authors:** Sharon Remmelzwaal, Adriana J. van Ballegooijen, Linda J. Schoonmade, Elisa Dal Canto, M. Louis Handoko, Michiel T. H. M. Henkens, Vanessa van Empel, Stephane R. B. Heymans, Joline W. J. Beulens

**Affiliations:** 1 Department of Epidemiology & Data Science, Amsterdam UMC, Vrije Universiteit Amsterdam, Amsterdam Cardiovascular Sciences, VU University Medical Centre, De Boelelaan 1089a, 1081HV, Amsterdam, The Netherlands; 2 Department of Nephrology, Amsterdam UMC, Vrije Universiteit Amsterdam, Amsterdam Cardiovascular Sciences, Amsterdam, The Netherlands; 3 University Library, VU, Amsterdam, The Netherlands; 4 Department of Cardiology, Amsterdam UMC, Vrije Universiteit Amsterdam, Amsterdam, The Netherlands; 5 Department of Cardiology, Maastricht University Medical Centre, Maastricht, The Netherlands; 6 Department of Cardiology, CARIM School for Cardiovascular Diseases Faculty of Health, Medicine and Life Sciences, Maastricht University, Maastricht, The Netherlands; 7 Julius Centre for Health Sciences and Primary Care, University Medical Centre Utrecht, Utrecht, The Netherlands

**Keywords:** Diastolic dysfunction, Heart failure with preserved ejection fraction, Natriuretic peptides, Systematic review, Diagnostic test

## Abstract

**Background:**

An overview of the diagnostic performance of natriuretic peptides (NPs) for the detection of diastolic dysfunction (DD) and heart failure with preserved ejection fraction (HFpEF), in a non-acute setting, is currently lacking.

**Methods:**

We performed a systematic literature search in PubMed and Embase.com (May 13, 2019). Studies were included when they (1) reported diagnostic performance measures, (2) are for the detection of DD or HFpEF in a non-acute setting, (3) are compared with a control group without DD or HFpEF or with patients with heart failure with reduced ejection fraction, (4) are in a cross-sectional design. Two investigators independently assessed risk of bias of the included studies according to the QUADAS-2 checklist. Results were meta-analysed when three or more studies reported a similar diagnostic measure.

**Results:**

From 11,728 titles/abstracts, we included 51 studies. The meta-analysis indicated a reasonable diagnostic performance for both NPs for the detection of DD and HFpEF based on AUC values of approximately 0.80 (0.73–0.87; *I*^2^ = 86%). For both NPs, sensitivity was lower than specificity for the detection of DD and HFpEF: approximately 65% (51–85%; *I*^2^ = 95%) versus 80% (70–90%; *I*^2^ = 97%), respectively. Both NPs have adequate ability to rule out DD: negative predictive value of approximately 85% (78–93%; *I*^2^ = 95%). The ability of both NPs to prove DD is lower: positive predictive value of approximately 60% (30–90%; *I*^2^ = 99%).

**Conclusion:**

The diagnostic performance of NPs for the detection of DD and HFpEF is reasonable. However, they may be used to rule out DD or HFpEF, and not for the diagnosis of DD or HFpEF.

## Background

Heart failure (HF) is a major public health problem with high morbidity and mortality [1, 2]. Over half of the HF patients have heart failure with preserved ejection fraction (HFpEF), which is featured by elevated left ventricular filling pressure during exercise despite a normal ejection fraction [3]. As HFpEF is a syndrome with different underlying pathophysiologic mechanisms, detection is difficult with current guidelines [3, 4].

The use of natriuretic peptides (NPs), specifically brain natriuretic peptide (BNP) and N-terminal prohormone of brain natriuretic peptide (NT-proBNP), is advised in HFpEF guidelines [3, 5, 6]. Detection of HFpEF in the stable outpatient population is difficult since levels of NPs are usually low as opposed to patients with heart failure with reduced ejection fraction (HFrEF) [7]. Compared with cut-offs for NPs in current guidelines [3, 5, 6], up to one-third of all HFpEF outpatients have NP levels below the typical diagnostic thresholds and will be missed [8, 9]. This is alarming as obesity is a common comorbidity in HFpEF patients and is associated with lower NT-proBNP levels, which could lead to underdiagnosis of HFpEF [10–12].

Although the exact underlying mechanism of HFpEF is still unclear, diastolic dysfunction (DD) is an established precursor in the development of HFpEF [13, 14]. NPs are not included in the current guidelines for the detection of DD [15]. However, emerging evidence suggests that BNP is higher in patients with DD in comparison to adults without DD [7, 16, 17]. Moreover, it has been demonstrated that levels gradually rise in parallel to the severity of diastolic abnormalities as assessed by echocardiography [18, 19]. As NPs are secreted by the ventricular walls as a result of abnormal preload and afterload, and systemic inflammation is apparent in patients with DD, NPs could be useful in detecting DD [20, 21]. Nonetheless, the performance of NPs for the detection of early subclinical DD is not as good as symptomatic DD [22].

Since early detection of DD and HFpEF in the non-acute setting is important for prevention and treatment strategies, a good diagnostic marker, such as NPs, is needed. However, a clear overview of the diagnostic performance of NPs for the detection of DD and HFpEF, in a non-acute setting, is currently lacking.

Therefore, this study aimed to systematically review and meta-analyse studies investigating the diagnostic performance of NPs for the detection of DD and HFpEF.

## Methods

### Data search

We performed a systematic review of PubMed and Embase.com from their inception to May 13, 2019 (SR and LS), according to the PRISMA-DTA statement [23]. The search terms ‘heart failure’ or ‘diastolic dysfunction’ were combined with general search terms ‘diagnostic performance’ or ‘markers’, as this broad search string was used for a set of systematic reviews describing a range of diagnostic markers (NPs, echo markers or biomarkers) (see Additional file 1). Reference lists of the identified articles were hand-searched for relevant publications. The protocol and search strategy were preregistered on PROSPERO (Registration number CRD42018065018). Because the protocol and search strategy were focussed on a broader research question, the identified studies were reported in three manuscripts focussed on echo parameters [24], biomarkers [25] and natriuretic peptides in this manuscript.

### Patient and public involvement

Patients were not involved in the generation of this meta-analysis.

### Study selection

Two reviewers independently screened titles, abstracts and full-text (SR/AJvB/MLH/JWJB). Studies were included if they (i) studied a diagnostic performance measure, (ii) studied the performance of NPs for the detection of DD and/or HFpEF, (iii) included a control population without DD or HFpEF or with HFrEF, (iv) had a cross-sectional study design (maximum follow-up 2 years) and (v) were written in English or Dutch. We excluded studies if they (i) studied the performance of the diagnostic marker for the detection of acute HF, (ii) are in rare patient populations (e.g. beta-thalassemia, hypertrophic cardiomyopathy or infiltrative disorders) or (iii) used a single echo marker as reference standard. Inconsistencies in study selection were resolved through consensus with a third reviewer (AJvB/JWJB). The mean positive and negative proportion of agreement between the reviewers for title/abstract screening was 40 and 96%, respectively. For full-text screening, the mean positive and negative proportion of agreement was 55 and 60%, respectively. For further details on the literature search and inclusion and exclusion criteria, see eMethods in Additional file 1.

### Data extraction

One reviewer (SR) extracted the data, including measures of study design, study population, number of participants, markers, diagnostic performance measures and demographics, which was appraised by a second reviewer (AJvB/JWJB).

### Quality assessment

Two reviewers (SR/AJvB/JWJB) independently evaluated the quality of the included studies using the QUADAS-2 checklist [26]. This checklist provides a quality score on four domains: patient selection, index test, reference standard and patient flow and timing. Each domain received a low, high or unclear risk of bias or concerns regarding applicability. A domain was rated as high risk of bias when one of the two or two of the three support questions were answered in a negative manner. A domain was rated as low risk of bias when two of the three or all support questions were answered in a positive manner. A domain was rated as unclear when one the of support questions could not be answered due to lack of information in the study. Inconsistencies in the quality assessment were resolved through consensus with a third reviewer (AJvB/JWJB). The mean positive and negative proportion of agreement between reviewers was 91 and 79%, respectively. For further details on the quality assessment, see eMethods in Additional file 1.

### Data synthesis

Studies were meta-analysed using a random-effects model when two or more studies investigated the same diagnostic measure in similar study populations with similar control populations and reported similar diagnostic performance measures. The studies had to provide confidence intervals of this diagnostic performance measure or sufficient information (2 × 2 table) to compute these confidence intervals. Forest plots of random-effects meta-analysis models were fitted to respectively AUCs, or sensitivities and specificities for all studies and stratified for cross-sectional versus case-control studies. In subgroup analyses, we examined differences by study design (cross-sectional versus case-control), geographic location (European versus other studies), assays (Roche versus other for NT-proBNP and FEIA versus RIA for BNP) and decade of publication (2000–2009 versus 2010–2019). Subgroup effect statistic was calculated by means of a Wald test to determine differences between respective subgroups, if both subgroups included a minimum of two studies [27]. In sensitivity analyses, we further determined heterogeneity by geographic location by including European studies only, or by study population by excluding studies with hospitalized patients. Heterogeneity was tested using *I*^2^ with *I*^2^ > 50% considered as substantial. Publication bias was evaluated by visual inspection of funnel plots. All analyses and plots were performed in RStudio 3.4.2 using the Metafor package [28]. Trivariate generalized mixed models (GLMM) were fitted in SAS Studio to meta-analyse PPVs and NPVs for all cross-sectional studies to account for differences in the prevalence of DD [29]. Percentage positive and negative agreement was calculated for title/abstract and full-text screening and quality assessment to determine the inter-rater reliability [30].

## Results

### Search results

From 11,728 titles/abstracts, 352 full-text articles were screened and 51 studies were included in the data extraction (Additional file 3: Figure S1). Twenty-three studies reported the diagnostic performance for the detection of DD and 27 studies for the detection of HFpEF and one study for both. Two studies reported diagnostic performance of ANP [20, 31].

### Study characteristics

Thirty studies were cross-sectional [18, 20, 22, 32–58] and twenty-one [31, 59–78] were case-control studies (Tables 1 and 2). Twenty-eight were performed in Europe [20, 32–36, 38, 40, 41, 43–45, 50, 52, 53, 55–57, 59, 63, 64, 66, 69, 73, 76–78], 11 in North America [22, 37, 39, 46, 49, 54, 65, 70, 72, 74, 75], 11 in Asia [31, 42, 47, 51, 58, 60–62, 67, 68, 71] and one was a multi-country study [48]. The control populations were either healthy controls (*N* = 14) [31, 35, 59, 60, 62, 65, 67–69, 71, 72, 75–77] or were recruited at the same department as the patient population, but without DD or HFpEF (*N* = 37) [32–34, 36–42, 58, 61, 63, 64, 66, 70]. The mean age of the DD and HFpEF populations ranged from 51 to 74 years and from 50.3 to 84.3, respectively. The mean age of the control populations ranged from 41.7 to 83.6 years. Five DD studies included patients with HFpEF [38, 43, 48, 73, 76], one DD study included patients with signs and symptoms [44], but did not exclude on LVEF, and eight DD studies only included patients with LVEF above 45 or 50% [18, 20, 45, 47, 50, 52, 53, 77]. The reference diagnoses used in the included studies varied from use of maximal velocity of the E-wave (e’) (*N* = 10) [43–45, 49, 50, 52, 54, 56, 57, 74] versus no tissue Doppler (TDI) measures (*N* = 14) [18, 20, 22, 38, 46–48, 51, 53, 55, 73, 76–78] for DD studies and, use of extensive criteria (*N* = 13) [31, 32, 34, 35, 38, 41, 59, 61, 63, 64, 66, 69, 72], signs and symptoms of HF in combination with LVEF (*N* = 11) [36, 40, 42, 58, 60, 62, 65, 67, 68, 70, 71], expert opinion (*N* = 1) [33] or catheterization (*N* = 3) [37, 39, 75] for HFpEF studies (Additional file 2: Table S1). Diagnostic performance of NPs for all studies for detection of either DD or HFpEF can be found in Additional file 2: Table S2.

**Table 1:** Study characteristics of the 28 included studies for the detection of heart failure with preserved ejection fraction

Author, country [Ref.]	Study design	Study setting	Marker (assay)	Patient population	Control population	Age and sex	
						Patient	Control
Heart failure with preserved ejection fraction							
Arques, EU [33]	Cross-sectional	Secondary	BNP (FEIA)	Hospitalised AF and DOE patients (*N* = 22)	Hospitalised AF patients with NCD (*N* = 19)	84.3 ± 5.2 y; 68% F	83.6 ± 5.1 y; 47% F
Arques, EU [32]	Cross-sectional	Secondary	BNP (FEIA)	AMB DOE Cath referrals (*N* = 15)	AMB DOE Cath referrals (*N* = 11)	58 (48–67) y; 27% F	57 (54–66) y; 55% F
Borlaug, USA [37]	Cross-sectional	Tertiary	BNP (unknown)	AMB Cath referrals (*N* = 32)	AMB Cath referrals with NCD (*N* = 23)	65 ± 13 y; 72% F	47 ± 17 y; 65% F
Martos, EU [38]	Cross-sectional	Tertiary	BNP (FEIA)	AMB HTN patients (*N* = 33)	AMB HTN patients (*N* = 20)	72 ± 11 y; 47% F	64 ± 10 y; 25% F
Mason, EU [63]	Case-control	Primary	BNP (FEIA) and NT-proBNP	Care home residents (*N* = 57)	Care home residents (*N* = 308)	84.2 ± 7.2 y; 74% F*	
Watson, EU [64]	Case-control	Tertiary	BNP (FEIA)	AMB patients (*N* = 75)	AMB HFrEF patients (*N* = 75)	75 ± 7 y; 41% F	70 ± 11 y; 27% F
Zordoky, CA [65]	Case-control	Tertiary	BNP (FEIA) and NT-proBNP	AMB patients (*N* = 24)	Healthy controls (*N* = 38)	67.5 (17.3) y; 25% F	61.5 (15.3) y; 52.6% F
Baessler, EU [34]	Cross-sectional	Tertiary	NT-proBNP	Obese patients (*N* = 88)	Obese patients (*N* = 119)	50.3 ± 7.3 y; 55% F	41.7 ± 12.1 y; 73% F
Barroso, EU [35]	Cross-sectional	Secondary	NT-proBNP	AMB patients (*N* = 77)	AMB controls (*N* = 55)	73 (68–77) y; 59.7% F	54 (48–61) y; 37.3% F
Berezin, EU [36]	Cross-sectional	Secondary	NT-proBNP	Hospitalised suspected HF patients (*N* = 79)	Hospitalised HFrEF patients (*N* = 85)	54.8 ± 6.6 y; 53.2% F	57.5 ± 6.7 y; 42.4% F
Celik, EU [59]	Case-control	Tertiary	NT-proBNP	AMB DHF patients (*N* = 71)	Controls (*N* = 50)	57.1 ± 7.4 y; 63.4% F	56.2 ± 7.0 y; 58% F
Cui, AS [60]	Case-control	Secondary	NT-proBNP	AMB patients (*N* = 172)	Random AMB controls from same hospital (*N* = 30)	73 ± 9.2 y; 55.8% F	67 ± 4.8 y; 40% F
Cui, AS [31]	Case-control	Secondary	NT-proBNP (ELISA) and MRproANP	Hospitalised patients (*N* = 65)	Age- and sex-matched hospitalised CVD patients (*N* = 75)	69 ± 14 y; 50.8% F	66 ± 11 y; 50.7% F
Kim, AS [61]	Case-control	Tertiary	NT-proBNP	AMB patients with DD (*N* = 228)	AMB patients with silent DD (*N* = 180)	68.3 ± 11.4 y; 61% F	62.3 ± 12.2 y; 58.3% F
Liu, AS [62]	Case-control	Tertiary	NT-proBNP (ELISA)	AMB chronic HFpEF patients (*N* = 50)	Healthy controls (*N* = 50)	64.3 ± 5.7 y; 46% F	63.8 ± 6 y; 54% F
Nikolova, USA [75]	Case-control	Tertiary	NT-proBNP	AMB patients (*N* = 52)	Healthy controls (*N* = 52) and controls with HF risk factor (*N* = 52)	57 ± 15 y; 37% F	Healthy/HF risk factor:
							52 ± 6 y; 37% F
							52 ± 9 y; 37% F
Polat, EU [66]	Case-control	Tertiary	NT-proBNP (ELISA)	AMB HFpEF patients (*N* = 44)	AMB patients without HF (*N* = 38)	60 ± 6.8 y; 45.5% F	57 ± 9 y; 47.4% F
Reddy, USA [39]	Cross-sectional	Tertiary	NT-proBNP	AMB HFpEF patients (*N* = 267)	AMB patients with NCD (*N* = 147)	68 ± 11 y; 61% F	56 ± 15 y; 59% F
Sanders-van Wijk, EU [40]	Cross-sectional	Secondary	NT-proBNP	AMB patients (*N* = 112)	AMB HFrEF patients (*N* = 458)	80 ± 7 y; 64% F	76 ± 7 y; 33% F
Santhanakrishnan, AS [67]	Case-control	Tertiary	NT-proBNP	AMB HF patients (*N* = 50)	Healthy controls (*N* = 50)	69 ± 12 y; 42% F	63 ± 8 y; 54% F
Shuai, AS [68]	Case-control	Tertiary	NT-proBNP	AMB patients (*N* = 45)	AMB HTN patients and healthy controls (*N* = 53)	68 ± 12 y; 55% F	Not reported
Sinning, EU [41]	Cross-sectional	Primary	NT-proBNP	Random residents (*N* = 70)	Random residents with HFrEF(*N* = 38)	67 (62–72) y; 50% F	64 (57.8–70.0) y; 21.1% F
Stahrenberg, EU [69]	Case-control	Primary	NT-proBNP	AMB CHF patients (*N* = 85)	Healthy controls (*N* = 188)	Not reported	56 (52–63) y; 66% F
Toma, CA [70]	Case-control	Tertiary	NT-proBNP (RAMP)	AMB patients (*N* = 21)	AMB HFrEF patients (*N* = 48)	70 (16) y; 52.4% F	66 (13.7) y; 27.1% F
Wang, AS [42]	Cross-sectional	Tertiary	NT-proBNP	AMB HTN patients (*N* = 68)	AMB HTN patients (*N* = 39)	68 ± 10 y; 54.1% F	60 ± 12 y; 33.3% F
Wong, AS [71]	Case-control	Tertiary	NT-proBNP	AMB HF patients (*N* = 30)	Healthy controls (*N* = 30)	64.1 ± 9.1 y	65.9 ± 6.7 y
Wong, AS [58]	Cross-sectional	Tertiary	NT-proBNP	Cohort 1 + 2: AMB patients (*N* = 68 + *N* = 179)	Cohort 1 + 2: AMB HFrEF patients (*N* = 115 + *N* = 145)	Cohort 1/2:	Cohort 1/2:
						65.9 ± 12.9 y; 37.5% F	57.1 ± 11.1 y; 18.2% F
							70.3 ± 11.0 y; 16.6% F
						76.6 ± 9.0 y; 46.4% F	
Zile, USA [72]	Case-control	Primary and tertiary	NT-proBNP (ChLIA)	AMB patients (*N* = 61)	Healthy controls (*N* = 241)	66 ± 1 y; 59% F	58 ± 1 y; 70% F

Age depicted in mean ± standard deviation, median (IQR) or median (minimum-maximum)

*EU* Europe, *AS* Asia, *CA* Canada, *y* years, *pro-ANP* pro-atrial natriuretic peptide, *BNP* brain natriuretic peptide, *AMB* ambulatory, *F* female, *AF* atrial fibrillation, *DD* diastolic dysfunction, *FEIA* fluorescence immunoassay, *HF* heart failure, *NT-proBNP* N-terminal prohormone of brain natriuretic peptide, *HFpEF* heart failure with preserved ejection fraction, *HTN* hypertension, *DOE* dyspnoeic on exertion, *HFrEF* heart failure with reduced ejection fraction, *NCD* non-cardiac dyspnoea, *Cath* catheterization, *CHF* chronic heart failure, *DHF* diastolic heart failure, *ChLIA* chemiluminescence immunoassay

*Reported for total study population

**Table 2:** Study characteristics of the 24 included studies for the detection of diastolic dysfunction

Author, country [Ref.]	Study design	Study setting	Marker (assay)	Patient population	Control population	Age and sex	
						Patient	Control
Diastolic dysfunction							
Bakowski, EU [20]	Cross-sectional	Secondary	ANP and BNP (RIA)	AMB patients with AF (*N* = 27)	AMB patients with AF (*N* = 15)	58.6 ± 8.2 y; 45.2% F*	
Bettencourt, EU [76]	Case-control	Tertiary	BNP (RIA)	AMB referrals (*N* = 17)	Healthy controls (*N* = 9)	71 (11) y; 52.9% F	65 (7) y; 44.4% F
Crowson, USA [74]	Case-control	Primary	BNP (FEIA)	Non-RA participants (*N* = 82)	Non-RA participants (*N* = 1455)	**Non-RA:** 61.3 ± 10.1 y; 55%F*	
Goto, AS [47]	Cross-sectional	Secondary	BNP (RIA)	AMB CAD patients with AP (*N* = 91)	AMB CAD patients with AP (*N* = 189)	67.4 ± 8.2 y; 18.7% F	66.3 ± 8.5 y; 30.7% F
Grewal, CA + EU [48]	Cross-sectional	Primary	BNP (RIA) and NT-proBNP	AMB HFpEF patients (*N* = 72)	AMB HFpEF patients (*N* = 109)	70 ± 10 y; 35% F	65 ± 12 y; 35% F
Karaca, EU [77]	Case-control	Tertiary	BNP (FEIA)	AMB HTN patients (*N* = 30)	Healthy controls (*N* = 20)	53 ± 9.2 y; 40% F	44 ± 4.8 y; 45% F
Lukowicz, EU [78]	Case-control	Primary	BNP (RIA)	General population (*N* = 38)	General population with SD (*N* = 16)	57 ± 10 y; 42% F	57 ± 4 y; 38% F
Mak, NA [49]	Cross-sectional	Tertiary	BNP (FEIA)	Echo referrals (*N* = 16)	Echo referrals (*N* = 84)	64 ± 13 y; 3% F*	
Mannacio, EU [50]	Cross-sectional	Tertiary	BNP (FEIA)	Hospitalised patients (*N* = 25)	Hospitalised patients (*N* = 14)	67 ± 8.6 y; 31.9% F*	
Martos, EU [38]	Cross-sectional	Tertiary	BNP (FEIA)	AMB HTN patients (*N* = 33)	AMB HTN patients (*N* = 20)	68 ± 9 y; 24% F	64 ± 10 y; 25% F
Redfield, USA [22]	Cross-sectional	Primary	BNP (FEIA)	Random residents (*N* = 137)	Random residents (*N* = 1817)	**Random:** 62 ± 11 y; 52% F*	
Wei, AS [51]	Cross-sectional	Secondary	BNP (FEIA)	AMB HTN patients (*N* = 61)	AMB HTN patients (*N* = 74)	70 ± 10 y; 32% F	66 ± 13 y; 35% F
Zapata, EU [52]	Cross-sectional	Secondary	BNP (FEIA) and NT-proBNP	ICU patients (*N* = 50)	ICU patients (*N* = 36)	**Mild DD:** 68 ± 11 y; 51% F	57 ± 13 y; 19% F
						**Moderate DD:** 72 ± 5 y, 17% F	
						**Severe DD:** 69 ± 9 y; 33% F	
Zuber, EU [53]	Cross-sectional	Tertiary	BNP (FEIA)	AMB patients (*N* = 7)	AMB patients (*N* = 129)	64 (range 19–88) y*	
Ansari, EU [43]	Cross-sectional	Tertiary	NT-proBNP	AMB HFpEF patients (*N* = 11)	AMB HFpEF patients (*N* = 14)	65 (range:22–97) y; 49% F*	
Barragan, EU [44]	Cross-sectional	Tertiary	NT-proBNP	AMB DOE patients (*N* = 45)	AMB DOE patients (*N* = 37)	61.4 ± 12.7 y; 87% F	43.2 ± 13.2 y; 59% F
Barutçuoğlu, EU [45]	Cross-sectional	Tertiary	NT-proBNP	AMB HTN patients (*N* = 122)	AMB HTN patients (*N* = 119)	55 ± 14 y; 61% F	53 ± 18 y; 67% F
Corteville, USA [46]	Cross-sectional	Tertiary	NT-proBNP	AMB CHD patients (*N* = 78)	AMB CHD patients (*N* = 652)	**< 100 pg/mL NT-proBNP:** 62 ± 10 y; 19% F*	
						**100–500 pg/mL NT-proBNP:** 68 ± 10 y; 21% F*	
						**> 500 pg/mL NT-proBNP:** 73 ± 10 y; 11% F*	
Kasner, EU [73]	Case-cohort	Tertiary	NT-proBNP	AMB HFpEF patients (*N* = 107)	AMB patients with chest discomfort (*N* = 73)	53 (17) y; 40% F	51 (16) y; 43% F
Mishra, USA [54]	Cross-sectional	Tertiary	NT-proBNP	AMB CKD patients (*N* = 284)	AMB CKD patients (*N* = 2384)	Quartiles of NT-proBNP	
						**Q1:** 54 (12) y; 35% F*	
						**Q4:** 62 (10) y; 47% F*	
Mocan, EU [55]	Cross-sectional	Secondary	NT-proBNP (ELISA)	Hospitalised MetS patients (*N* = 47)	Hospitalised MetS patients (*N* = 25)	59.2 ± 5.3 y; 64% F*	
Ravassa, EU [56]	Cross-sectional	Primary	NT-proBNP (EIA)	General population (*N* = 186)	General population (*N* = 541)	51.3 ± 15.4 y; 51.2% F*	
Tekten, EU [57]	Cross-sectional	Tertiary	NT-proBNP	AMB HTN patients with DD grade 1 (*N* = 18) or grade 2 (*N* = 10)	AMB HTN patients (*N* = 12)	**Grade 1/2:** 52 ± 5 y; 67% F 54 ± 8 y; 60% F	51 ± 5 y; 67% F
Tschöpe, EU [18]	Cross-sectional	Tertiary	NT-proBNP	AMB DOE patients (*N* = 68)	AMB patients (*N* = 50)	51 ± 9 y; 46% F	49 ± 10 y; 44% F

Age depicted in mean ± standard deviation, median (IQR) or median (minimum-maximum)

*EU* Europe, *AS* Asia, *CA* Canada, *y* years, *ANP* atrial natriuretic peptide, *BNP* brain natriuretic peptide, *RIA* radioimmunoassay, *AMB* ambulatory, *F* female, *AF* atrial fibrillation, *DD* diastolic dysfunction, *FEIA* fluorescence immunoassay, *SD* systolic dysfunction, *RA* rheumatoid arthritis, *NT-proBNP* N-terminal prohormone of brain natriuretic peptide, *CAD* coronary artery disease, *AP* angina pectoris, *HFpEF* heart failure with preserved ejection fraction, *HTN* hypertension, *ICU* intensive care unit, *DOE* dyspnoeic on exertion, *CHD* coronary heart disease, *CKD* chronic kidney disease, *Q* quartile, *MetS* metabolic syndrome

*Reported for total study population

### Quality scores

The risk of bias for the domains patient selection, index test and reference standard was high: respectively 76, 53 and 61% (Additional file 3: Figure S2). This high risk of bias was mainly caused by a case-control design (*N* = 21), use of threshold values of NPs that were not pre-specified (*N* = 25) and inappropriate reference standards (*N* = 27). Only 20% of the studies had high risk of bias for the domain flow and timing. The funnel plots for NPs with DD and HFpEF suggest some evidence of publication bias for smaller studies (Additional file 3: Figure S3).

### Meta-analyses

**Fig. 1:**
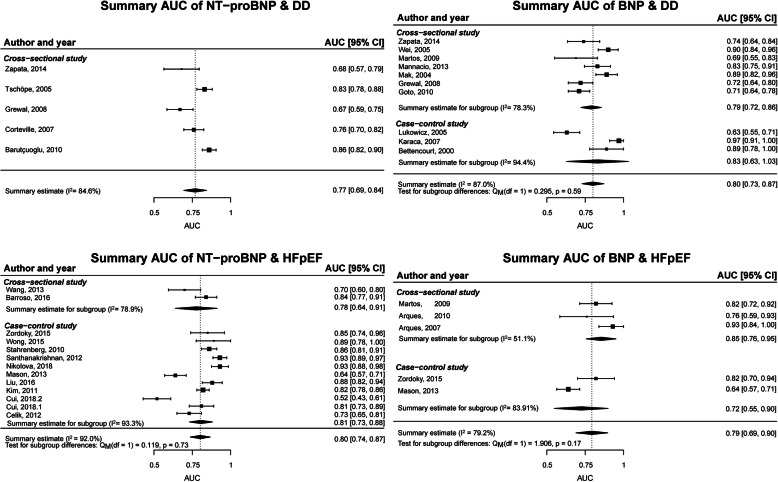
Meta-analysis of AUC values of NT-proBNP and BNP for the detection of DD with controls without DD or for HFpEF with controls without HFpEF

For detection of DD, AUC values of five NT-proBNP studies and ten BNP studies showed a summary estimate of 0.77 (0.69–0.84; *I*^2^ = 84.6%) and of 0.80 (0.73–0.87; *I*^2^ = 87.0%), respectively (Fig. 1). The AUC values for detection of HFpEF versus controls without HFpEF (Fig. 1) of 13 NT-proBNP studies showed a summary estimate of 0.80 (0.74–0.87; *I*^2^ = 92.1%) and 0.79 (0.69–0.90; *I*^2^ = 79.2%) for five BNP studies. Subgroup analyses did not show differences between cross-sectional and case-control studies for BNP for detection of DD and HFpEF and for NT-proBNP for detection of HFpEF (*p* values > 0.17). A summary estimate for cross-sectional studies for NT-proBNP for detection of HFpEF was not feasible, as only two studies were cross-sectional [35, 42]. Excluding case-control studies for BNP for the detection of HFpEF only resulted in a summary estimate of 0.85 (0.76–0.95; *I*^2^ = 51.1%) (Fig. 1). For the detection of HFpEF in comparison to HFrEF, AUC values of seven NT-proBNP studies showed a summary estimate of 0.69 (0.66–0.72; *I*^2^ = 0%). Excluding case-control studies did not alter the summary estimates and heterogeneity: mean AUC of 0.69 (0.66–0.72; *I*^2^ = 0%).

**Fig. 2:**
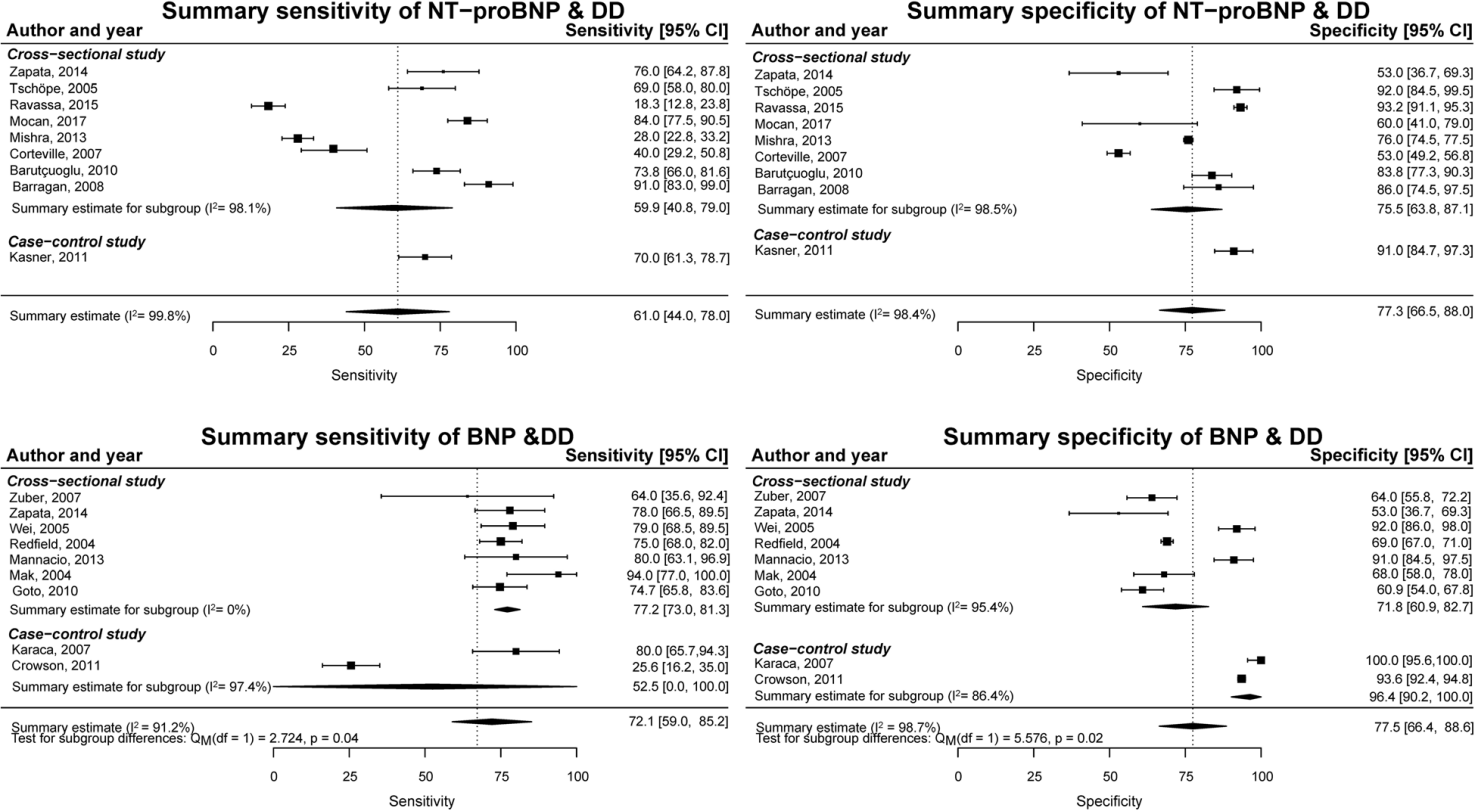
Meta-analysis of sensitivity and specificity of NT-proBNP and BNP for the detection of DD with controls without DD

For the detection of DD, nine studies reported sensitivity and specificity for NT-proBNP with a mean of 62% (44–80%; *I*^2^ = 98.0%) and a mean of 77% (67–88%; *I*^2^ = 98.4%), respectively. Nine studies reported this for BNP with a mean sensitivity of 72% (59–85%; *I*^2^ = 91.1%) and a mean specificity of 78% (67–87%; *I*^2^ = 98.7%) (Fig. 2). Subgroup analyses showed differences in sensitivity and specificity between cross-sectional and case-control studies for BNP for detection of DD (*p* values < 0.05) with mean sensitivities of 77% (73–81%; *I*^2^ = 0%) and 53% (0–100%; *I*^2^ = 97.4%) and mean specificities of 72% (61–83%; *I*^2^ = 95.4%) and 96% (90–100%; *I*^2^ = 86.4%), respectively. Excluding case-control studies did not reduce heterogeneity for NT-proBNP for detection of DD with non-invasive measures as a reference standard, mean sensitivity 61% (41–81%; *I*^2^ = 97.6%) and mean specificity 76% (64–87%; *I*^2^ = 99.1%). However, exclusion of case-control studies resulted in a mean sensitivity of 77% (73–81%; *I*^2^ = 0%) and mean specificity of 72% (61–83%; *I*^2^ = 95.4%) for BNP with inconsistent effects on heterogeneity.

**Fig. 3:**
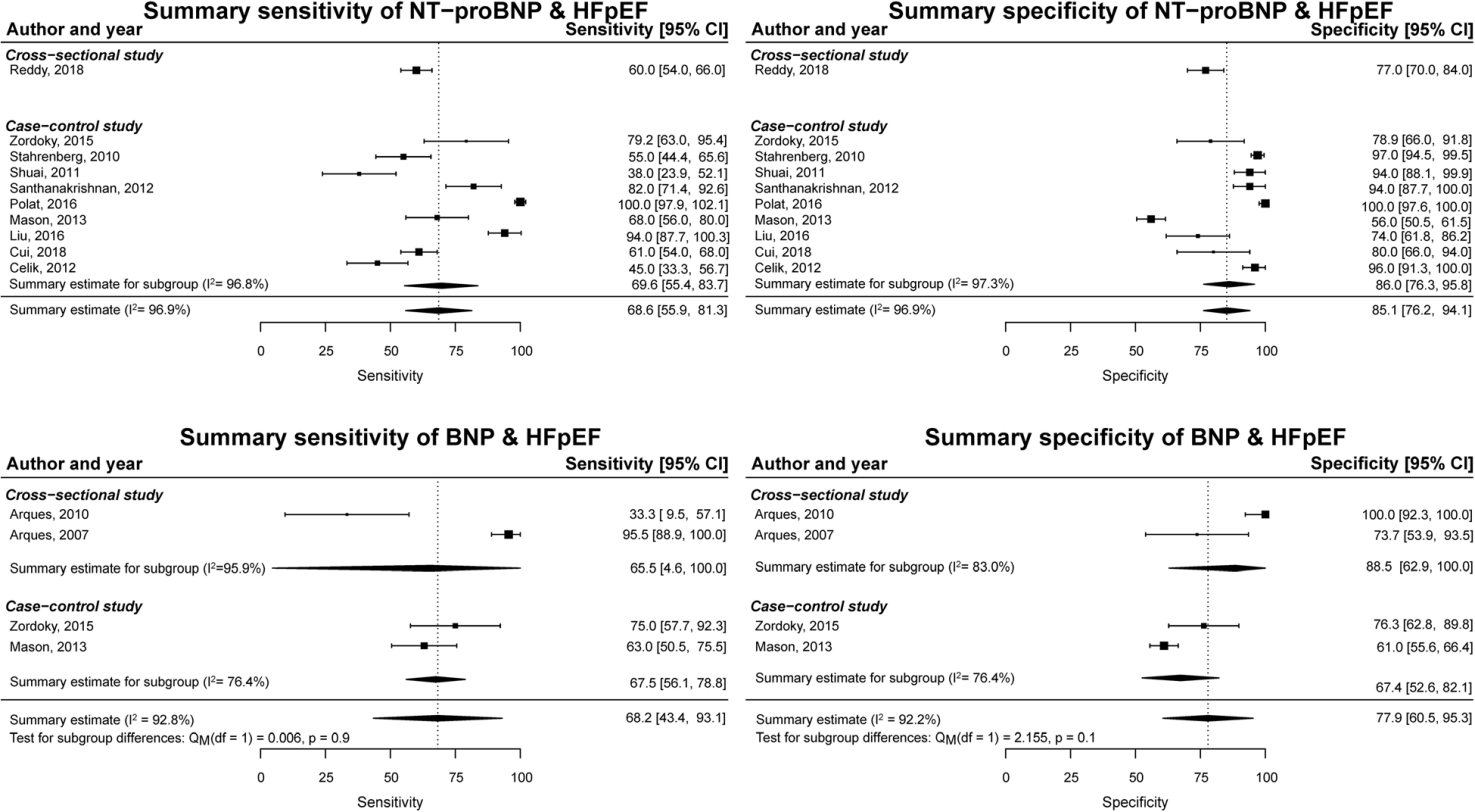
Meta-analysis of sensitivity and specificity of NT-proBNP and BNP for the detection of HFpEF with controls without HFpEF

Sensitivity and specificity were reported for NT-proBNP for detection of HFpEF in ten studies with a mean of 69% (56–81%; *I*^2^ = 96.9%) and a mean of 85% (76–91%; *I*^2^ = 98.9%), respectively. Four studies reported sensitivity and specificity for BNP for detection of HFpEF with a mean of 68% (44–93%; *I*^2^ = 92.8%) and a mean of 78% (61–95%; *I*^2^ = 92.2%), respectively (Fig. 3). With only one cross-sectional study for NT-proBNP, summary estimates could not be computed. Subgroup analyses did not show differences between cross-sectional and case-control studies for BNP for the detection of HFpEF (*p* values > 0.1).

### Subgroup analyses

Subgroup analyses did not show differences between European studies and studies from other countries (*p* values > 0.05), nor for BNP or NT-proBNP assay (*p* values > 0.07), nor for the decade of publication (2000–2009 versus 2010–2019) (*p* values > 0.08) (Additional file 2: Table S3).

#### Sensitivity analysis

Including only ambulatory patients (*N* = 3–12 studies) resulted in lower heterogeneity for BNP for detection of HFpEF with similar summary estimates: AUC of 0.75 (0.66–0.85; *I*^2^ = 69.7%).

### Positive and negative predictive values

**Fig. 4:**
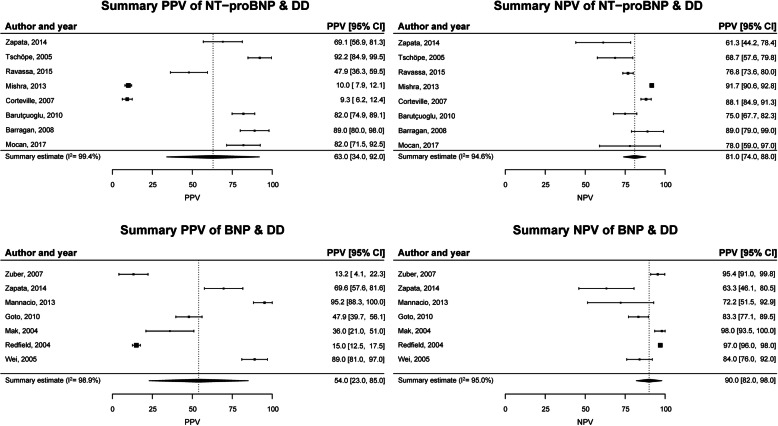
Meta-analysis of positive and negative predictive value of NT-proBNP and BNP for the detection of DD with controls without DD

For detection of DD, eight cross-sectional studies reported PPV and NPV for NT-proBNP with a mean of 63% (34–92%) and a mean of 81% (74–88%), respectively (Fig. 4). Seven cross-sectional studies reported this for BNP with a mean PPV of 54% (23–85%) and a mean NPV of 90% (82–98%). With only two cross-sectional studies for both NPs, summary estimates for detection of HFpEF could not be computed. The two NT-proBNP studies for the detection of HFpEF showed inconsistent results, but remarkably, for BNP, the PPV was higher than NPV: around 90 and 70%, respectively.

### Incremental diagnostic performance

Three studies reported incremental values of NPs on top of clinical models, but NPs did not improve the diagnostic performance of the model [34, 36, 72]. Two studies reported the diagnostic performance of clinical models including NPs, but the diagnostic performance of the model only was not reported [48, 56].

## Discussion

Our study is the first systematic review and meta-analysis of NPs as a diagnostic marker for the detection of DD and HFpEF. The meta-analysis indicates a reasonable diagnostic performance for both NPs for detection of DD and HFpEF with AUC values around 0.80, although heterogeneity between studies was high. Heterogeneity was partly explained by the case-control design of half of the BNP studies for detection of HFpEF. For both NPs, sensitivity was lower than specificity: approximately 65% versus 80%, respectively. Both NPs have adequate ability to rule out DD with a NPV of approximately 80%. The ability of both NPs to prove DD is lower with a PPV of approximately 60%. The risk of bias was generally high for three of the four domains.

Our systematic review and meta-analysis has several strengths. Our study provides a comprehensive overview of the diagnostic performance of NPs for detection of HFpEF and DD. The systematic review and meta-analysis are performed according to the PRISMA-DTA statement and included a quality assessment [23, 26]. However, this systematic review also has some limitations, such as substantial heterogeneity due to the quality of the included studies. The heterogeneity of included studies can be due to spectrum bias, which is a bias introduced by different inclusion criteria resulting in different study populations. We therefore performed sensitivity analyses excluding studies with hospitalized patients, but this did not affect our results. We also performed subgroup analyses for other characteristics such as geographical region, assay and decade of publication, but we did not detect any differences. However, study settings also differed across included studies in other aspects such as sex and age of the study population for which we could not perform a meta-analysis as the groups became too small. The effect of spectrum bias on the reported diagnostic measures is hard to quantify as it is unclear if this would lead to over- or underestimation of the diagnostic measures. Another explanation for the heterogeneity of included studies could be the heterogeneous nature of the HFpEF syndrome.

Overall, included studies had a high risk of bias with half of the studies using a case-control design. This results in an overestimation of diagnostic performance, as the contrast in clinical characteristics between patient and control population is large. Moreover, in case-control studies, the control population does not accurately reflect the population suspected to have DD or HFpEF, for whom NPs will be used in clinical practice. This limits the applicability of the results to other studies and clinical practice. Restricting the meta-analyses to cross-sectional studies substantially reduced heterogeneity (*I*^2^ = 51.1%) in studies for BNP for detection of HFpEF, but the summary estimates remained similar. For the meta-analyses for PPV and NPV, case-control studies were already excluded because of the use of prevalence estimates in Trivariate GLMM.

This study showed that the diagnostic performance of NPs for detection of DD and HFpEF, versus no DD or HFpEF, is reasonable with summary AUC values around 0.80 for both NT-proBNP and BNP, while the diagnostic performance of NT-proBNP for detection of HFpEF versus HFrEF was lower: around 0.70. For both NPs for the detection of DD and HFpEF, specificity (~ 80%) was higher than sensitivity (~ 60–70%). The overall performance persisted in analyses excluding case-control studies. Therefore, both for detection of DD versus no DD or HFpEF versus no HFpEF, our results indicate that these measures seem to perform better for ruling out of DD or HFpEF than for making the diagnosis. However, the specific performance of NPs in clinical practice also depends on the clinical setting. The high percentage of false-negatives might be more severe for secondary or tertiary care, while in primary care, NPs may be more important to rule out DD or HFpEF, for which other diagnostic characteristics, such as NPV, are important. Our results indicate that NPs are useful to rule out DD or HFpEF in primary care with a low prevalence of these conditions but are less suitable to use to differentiate HFpEF from HFrEF.

NT-proBNP and BNP have equal capability for ruling out or ruling in DD, but NT-proBNP has a higher specificity for detection of HFpEF. In general, based on this meta-analysis, one NP is not clearly preferred over the other for the detection of DD and HFpEF. The ranges of cut-off values used in the included studies were wide. In comparison to the current guidelines, only six studies used the same cut-off value for NT-proBNP as the new HFA-PEFF diagnostic algorithm [3]. Therefore, we recommend to use the cut-off values as proposed by the current guidelines [3, 6].

For detection of DD, both NPs have a substantially lower PPV (~ 60%) than NPV (~ 85%). This means that both NPs are potentially better in ruling out DD than proving DD. Current guidelines for detection of DD do not include NPs as a diagnostic marker [15]. NPs are released as a consequence of volume overload, a characteristic that is absent in (asymptomatic) DD. Consequently, the positive predictive value for the detection of DD is low and will result in misclassification, and therefore, our findings are in line with guidelines for the diagnosis of DD, as NPs are not advised to diagnose DD. However, the guidelines could provide room for the use of NPs to rule out DD in certain settings [15]. For example, in patients with exertional dyspnoea, NPs have a very good ability to diagnose DD [18]. Our study also provides evidence that NPs might be useful to rule out DD in specific settings wherein a low prevalence of DD occurs, such as primary care. This approach could be suitable for screening patients at high risk of HFpEF in primary care such as diabetes patients.

For the detection of HFpEF, a trend towards a higher PPV than NPV is observed, suggesting that BNP could be useful for diagnosing HFpEF instead of ruling out HFpEF. This is in contrast with guidelines that propose to use NPs only for ruling out HFpEF [5, 6]. In acute settings, the diagnostic performance of NPs is good to detect acute HF from non-cardiac dyspnoea, as NP levels are higher in patients with acute HF [79, 80]. In non-acute HFpEF patients, NP levels can be closer to normal than the acute setting. This makes it more difficult to distinguish HFpEF from non-HFpEF patients based on NPs, especially in combination with common comorbidities that complicate the diagnosis further [10, 81]. Therefore, NPs should be used in combination with echocardiography for initial diagnosis of HFpEF, as guidelines recommend [3]. Evidence of the incremental value of NPs on top of clinical characteristics or echocardiography measures is limited, but important. We therefore recommend future studies to compute incremental values of NPs on top of clinical or echocardiographic diagnostic models for the detection of HFpEF, confirmed by catheterization. Furthermore, future studies should aim to reduce bias by using cross-sectional studies with pre-specified cut-off values and correct reference diagnoses with transparent patient population selection procedures. As our study shows the ability of NPs to rule out DD and as earlier recognition of HFpEF is key to prevent late diagnosis, future studies should focus on the possibilities of NPs in screening programmes in patients at risk in primary care such as patients with type 2 diabetes, for detection of DD as precursor of HFpEF.

## Conclusion

This systematic review and meta-analysis of 51 studies shows that NPs have reasonable diagnostic performance for the detection of DD and HFpEF in a non-acute setting. NPs are useful to rule out DD and would not be a tool to rule in DD. NPs have value in the diagnosis of HFpEF, but not for ruling out HFpEF. However, NPs should be used in combination with echocardiography. As the risk of bias of the included studies is high and sensitivity of NPs for detection of DD or HFpEF is low compared to specificity, the use of NPs alone to detect DD or HFpEF should be discouraged as recommended by current guidelines. Nonetheless, the high NPV observed for both DD and HFpEF indicates they might be useful for screening of high-risk patients in primary care such as those with diabetes. For future research and guidelines, well-performed cross-sectional studies with pre-specified cut-off values for NPs are needed for unbiased estimates of diagnostic performance measures of NPs, especially for use in primary care.

## Supplementary information

**Additional file 1.** Search strategy for PubMed and Embase.com.

**Additional file 2: **Additional information about the methods. **Table S1.** Information of the reference diagnoses of the 51 included studies. **Table S2.** Diagnostic performance measures of the 51 included studies. **Table S3.** Subgroup analyses stratified by geographic location or assay.

**Additional file 3: Figure S1.** Prisma Flow diagram of the included studies. **Figure S2.** Risk of Bias score and applicability assessment of the 51 included studies. **Figure S3.** Funnel plots for summary estimates of NPs for the detection of DD and HFpEF.

## Data Availability

Data available on request.

## References

[1] McMurray JJV, Adamopoulos S, Anker SD (2012). ESC guidelines for the diagnosis and treatment of acute and chronic heart failure 2012. Eur J Heart Fail.

[2] Yancy Clyde W, Jessup M, Bozkurt B (2017). 2017 ACC/AHA/HFSA focused update of the 2013 ACCF/AHA guideline for the management of heart failure: a report of the American College of Cardiology/American Heart Association Task Force on clinical practice guidelines and the Heart Failure Society of America. Circulation.

[3] Pieske B, Tschöpe C, de Boer RA (2019). How to diagnose heart failure with preserved ejection fraction: the HFA–PEFF diagnostic algorithm: a consensus recommendation from the Heart Failure Association (HFA) of the European Society of Cardiology (ESC). Eur Heart J.

[4] Lam CS, Roger VL, Rodeheffer RJ (2009). Pulmonary hypertension in heart failure with preserved ejection fraction: a community-based study. J Am Coll Cardiol.

[5] Paulus WJ, Tschöpe C, Sanderson JE (2007). How to diagnose diastolic heart failure: a consensus statement on the diagnosis of heart failure with normal left ventricular ejection fraction by the Heart Failure and Echocardiography Associations of the European Society of Cardiology. Eur Heart J.

[6] Ponikowski P, Voors AA, Anker SD (2016). 2016 ESC guidelines for the diagnosis and treatment of acute and chronic heart failureThe Task Force for the diagnosis and treatment of acute and chronic heart failure of the European Society of Cardiology (ESC) developed with the special contribution of the Heart Failure Association (HFA) of the ESC. Eur Heart J.

[7] Jorge AL, Rosa MLG, Martins WA (2016). The prevalence of stages of heart failure in primary care: a population-based study. J Card Fail.

[8] Kasahara S, Sakata Y, Nochioka K, et al. Comparable prognostic impact of BNP levels among HFpEF, borderline HFpEF and HFrEF: a report from the CHART-2 study. Heart Vessel. 2018. 10.1007/s00380-018-1150-4. 10.1007/s00380-018-1150-4 29569034

[9] Anjan VY, Loftus TM, Burke MA (2012). Prevalence, clinical phenotype, and outcomes associated with normal B-type natriuretic peptide levels in heart failure with preserved ejection fraction. Am J Cardiol.

[10] Obokata M, Reddy YNV, Pislaru SV (2017). Evidence supporting the existence of a distinct obese phenotype of heart failure with preserved ejection fraction. Circulation.

[11] Stavrakis S, Pakala A, Thadani U (2013). Obesity, brain natriuretic peptide levels and mortality in patients hospitalized with heart failure and preserved left ventricular systolic function. Am J Med Sci.

[12] DeFilippi CR, Fink JC, Nass CM (2005). N-terminal pro-B-type natriuretic peptide for predicting coronary disease and left ventricular hypertrophy in asymptomatic CKD not requiring dialysis. Am J Kidney Dis.

[13] Kane GC, Karon BL, Mahoney DW (2011). Progression of left ventricular diastolic dysfunction and risk of heart failure. JAMA.

[14] Redfield MM, Jacobsen SJ, Burnett JC (2003). Burden of systolic and diastolic ventricular dysfunction in the community: appreciating the scope of the heart failure epidemic. JAMA.

[15] Nagueh SF, Marino P, Oh JK (2016). Recommendations for the evaluation of left ventricular diastolic function by echocardiography: an update from the American Society of Echocardiography and the European Association of Cardiovascular Imaging. Eur Heart J Cardiovasc Imaging.

[16] Grandi AM, Laurita E, Selva E (2004). Natriuretic peptides as markers of preclinical cardiac disease in obesity. Eur J Clin Investig.

[17] Germing A, Gotzmann M, Schikowski T (2011). High frequency of diastolic dysfunction in a population-based cohort of elderly women - but poor association with the symptom dyspnea. BMC Geriatr.

[18] Tschöpe C, Kašner M, Westermann D (2005). The role of NT-proBNP in the diagnostics of isolated diastolic dysfunction: correlation with echocardiographic and invasive measurements. Eur Heart J.

[19] Iwanaga Y, Nishi I, Furuichi S (2006). B-type natriuretic peptide strongly reflects diastolic wall stress in patients with chronic heart failure: comparison between systolic and diastolic heart failure. J Am Coll Cardiol.

[20] Bakowski D, Wozakowska-Kaplon B, Opolski G (2009). The influence of left ventricle diastolic function on natriuretic peptides levels in patients with atrial fibrillation. Pacing Clin Electrophysiol.

[21] Fang L, Ellims AH, Beale AL (2017). Systemic inflammation is associated with myocardial fibrosis, diastolic dysfunction, and cardiac hypertrophy in patients with hypertrophic cardiomyopathy. Am J Transl Res.

[22] Redfield Margaret M, Rodeheffer Richard J, Jacobsen Steven J (2004). Plasma brain natriuretic peptide to detect preclinical ventricular systolic or diastolic dysfunction. Circulation.

[23] McInnes MF, Moher D, Thombs BD (2018). Preferred reporting items for a systematic review and meta-analysis of diagnostic test accuracy studies: the prisma-dta statement. JAMA.

[24] Dal Canto E, Remmelzwaal S, van Ballegooijen AJ, et al. Diagnostic value of echocardiographic markers for diastolic dysfunction and heart failure with preserved ejection fraction. Heart Fail Rev. 2020. 10.1007/s10741-020-09985-1. 10.1007/s10741-020-09985-1 PMC8739319 32488580

[25] Henkens MTHM, Remmelzwaal S, Robinson EL, et al. Risk of bias in studies investigating novel diagnostic biomarkers for heart failure with preserved ejection fraction. A systematic review. Eur J Heart Fail. 2020;n/a(n/a). 10.1002/ejhf.1944. 10.1002/ejhf.1944 PMC7689920 32592317

[26] Whiting PF, Rutjes AW, Westwood ME (2011). Quadas-2: a revised tool for the quality assessment of diagnostic accuracy studies. Ann Intern Med.

[27] Rubio-Aparicio M, López-López JA, Viechtbauer W (2020). Testing categorical moderators in mixed-effects meta-analysis in the presence of heteroscedasticity. J Exp Educ.

[28] Viechtbauer W. Conducting meta-analyses in R with the metafor package. 2010;36(3):48. 10.18637/jss.v036.i03 [published Online First: 2010-08-05].

[29] Ma X, Nie L, Cole SR (2013). Statistical methods for multivariate meta-analysis of diagnostic tests: an overview and tutorial. Stat Methods Med Res.

[30] de Vet HCW, Mokkink LB, Terwee CB (2013). Clinicians are right not to like Cohen’s κ. BMJ.

[31] Cui K, Huang W, Fan J (2018). Midregional pro-atrial natriuretic peptide is a superior biomarker to N-terminal pro-B-type natriuretic peptide in the diagnosis of heart failure patients with preserved ejection fraction. Medicine (Baltimore).

[32] Arques S, Jaubert M-P, Bonello L (2010). Usefulness of basal B-type natriuretic peptide levels for the diagnosis of diastolic heart failure in young patients: An echocardiographic-catheterization study. Int J Cardiol.

[33] Arques S, Roux E, Sbragia P (2007). Usefulness of bedside tissue Doppler echocardiography and B-type natriuretic peptide (BNP) in differentiating congestive heart failure from noncardiac cause of acute dyspnea in elderly patients with a normal left ventricular ejection fraction and permanent, nonvalvular atrial fibrillation: insights from a prospective, monocenter study. Echocardiography.

[34] Baessler A, Strack C, Rousseva E (2012). Growth-differentiation factor-15 improves reclassification for the diagnosis of heart failure with normal ejection fraction in morbid obesity. Eur J Heart Fail.

[35] Barroso MC, Kramer F, Greene SJ (2016). Serum insulin-like growth factor-1 and its binding protein-7: potential novel biomarkers for heart failure with preserved ejection fraction. BMC Cardiovasc Disord.

[36] Berezin AE, Kremzer AA, Martovitskaya YV (2016). Pattern of endothelial progenitor cells and apoptotic endothelial cell-derived microparticles in chronic heart failure patients with preserved and reduced left ventricular ejection fraction. EBioMedicine.

[37] Borlaug BA, Nishimura RA, Sorajja P (2010). Exercise hemodynamics enhance diagnosis of early heart failure with preserved ejection fraction. Circ Heart Fail.

[38] Martos R, Baugh J, Ledwidge M (2009). Diagnosis of heart failure with preserved ejection fraction: improved accuracy with the use of markers of collagen turnover. Eur J Heart Fail.

[39] Reddy YNV, Carter RE, Obokata M (2018). A simple, evidence-based approach to help guide diagnosis of heart failure with preserved ejection fraction. Circulation.

[40] Sanders-van Wijk S, van Empel V, Davarzani N (2015). Circulating biomarkers of distinct pathophysiological pathways in heart failure with preserved vs. reduced left ventricular ejection fraction. Eur J Heart Fail.

[41] Sinning C, Kempf T, Schwarzl M (2017). Biomarkers for characterization of heart failure – distinction of heart failure with preserved and reduced ejection fraction. Int J Cardiol.

[42] Wang Y-C, Yu C-C, Chiu F-C (2013). Soluble ST2 as a biomarker for detecting stable heart failure with a normal ejection fraction in hypertensive patients. J Card Fail.

[43] Ansari U, Behnes M, Hoffmann J (2018). Galectin-3 reflects the echocardiographic grades of left ventricular diastolic dysfunction. Ann Lab Med.

[44] Barragán A, Lacalzada J, de la Rosa A (2008). Relationship between slightly elevated NT-proBNP and alterations in diastolic function detected by echocardiography in patients without structural heart disease. Int J Cardiol.

[45] Barutçuoǧlu B, Parildar Z, Başol G (2010). The detection of left ventricular diastolic dysfunction in hypertensive patients: performance of N-terminal probrain natriuretic peptide. Blood Press.

[46] Corteville DCM, Bibbins-Domingo K, Wu AHB (2007). N-terminal pro-B-type natriuretic peptide as a diagnostic test for ventricular dysfunction in patients with coronary disease: data from the heart and soul study. Arch Intern Med.

[47] Goto T, Ohte N, Wakami K (2010). Usefulness of plasma brain natriuretic peptide measurement and tissue Doppler imaging in identifying isolated left ventricular diastolic dysfunction without heart failure. Am J Cardiol.

[48] Grewal J, McKelvie R, Lonn E (2014). BNP and NT-proBNP predict echocardiographic severity of diastolic dysfunction. Eur J Heart Fail.

[49] Mak GS, DeMaria A, Clopton P (2004). Utility of B-natriuretic peptide in the evaluation of left ventricular diastolic function: comparison with tissue Doppler imaging recordings. Am Heart J.

[50] Mannacio V, Antignano A, De Amicis V (2013). B-type natriuretic peptide as a biochemical marker of left ventricular diastolic function: assessment in asymptomatic patients 1 year after valve replacement for aortic stenosis. Interact Cardiovasc Thorac Surg.

[51] Wei T, Zeng C, Chen L (2005). Bedside tests of B-type natriuretic peptide in the diagnosis of left ventricular diastolic dysfunction in hypertensive patients. Eur J Heart Fail.

[52] Zapata L, Betbese AJ, Roglan A (2014). Use of B-type natriuretic peptides to detect the existence and severity of diastolic dysfunction in non-cardiac critically ill patients: a pilot study. Minerva Anestesiol.

[53] Zuber M, Kipfer P, Attenhofer Jost CH (2007). Usefulness of acoustic cardiography to resolve ambiguous values of B-type natriuretic peptide levels in patients with suspected heart failure. Am J Cardiol.

[54] Mishra RK, Li Y, Ricardo AC (2013). Association of N-terminal pro-B-type natriuretic peptide with left ventricular structure and function in chronic kidney disease (from the Chronic Renal Insufficiency Cohort [CRIC]). Am J Cardiol.

[55] Mocan M, Anton F, Suciu S, et al. Multimarker assessment of diastolic dysfunction in metabolic syndrome patients. Metab Syndr Relat Disord. 2017;15. 10.1089/met.2017.0060. 10.1089/met.2017.0060 29099655

[56] Ravassa S, Kuznetsova T, Varo N (2015). Biomarkers of cardiomyocyte injury and stress identify left atrial and left ventricular remodelling and dysfunction: A population-based study. Int J Cardiol.

[57] Tekten T, Yenisey C, Ceyhan C (2007). Correlation between N-terminal pro B-natriuretic peptide and ultrasonic backscatter: implications for diastolic dysfunction in hypertension. Echocardiography.

[58] Wong LL, Zou R, Zhou L (2019). Combining circulating microRNA and NT-proBNP to detect and categorize heart failure subtypes. J Am Coll Cardiol.

[59] Celik A, Koc F, Kadi H (2012). Relationship between red cell distribution width and echocardiographic parameters in patients with diastolic heart failure. Kaohsiung J Med Sci.

[60] Cui Y, Qi X, Huang A (2018). Differential and predictive value of galectin-3 and soluble suppression of tumorigenicity-2 (sST2) in heart failure with preserved ejection fraction. Med Sci Monit.

[61] Kim H, Yoon H-J, Park H-S (2011). Usefulness of tissue Doppler imaging-myocardial performance index in the evaluation of diastolic dysfunction and heart failure with preserved ejection fraction. Clin Cardiol.

[62] Liu S, Iskandar R, Chen W (2016). Soluble glycoprotein 130 and heat shock protein 27 as novel candidate biomarkers of chronic heart failure with preserved ejection fraction. Heart Lung Circ.

[63] Mason JM, Hancock HC, Close H (2013). Utility of biomarkers in the differential diagnosis of heart failure in older people: findings from the heart failure in care homes (HFinCH) diagnostic accuracy study. PLoS One.

[64] Watson CJ, Gupta SK, O'Connell E (2015). MicroRNA signatures differentiate preserved from reduced ejection fraction heart failure. Eur J Heart Fail.

[65] Zordoky BN, Sung MM, Ezekowitz J (2015). Metabolomic fingerprint of heart failure with preserved ejection fraction. PLoS One.

[66] Polat V, Bozcali E, Uygun T (2016). Diagnostic significance of serum galectin-3 levels in heart failure with preserved ejection fraction. Acta Cardiol.

[67] Santhanakrishnan R, Chong JPC, Ng TP (2014). Growth differentiation factor 15, ST2, high-sensitivity troponin T, and N-terminal pro brain natriuretic peptide in heart failure with preserved vs. reduced ejection fraction. Eur J Heart Fail.

[68] Shuai X-X, Chen Y-Y, Lu Y-X (2014). Diagnosis of heart failure with preserved ejection fraction: which parameters and diagnostic strategies are more valuable?. Eur J Heart Fail.

[69] Stahrenberg R, Edelmann F, Mende M (2010). The novel biomarker growth differentiation factor 15 in heart failure with normal ejection fraction. Eur J Heart Fail.

[70] Toma M, Mak GJ, Chen V (2017). Differentiating heart failure phenotypes using sex-specific transcriptomic and proteomic biomarker panels. ESC Heart Fail.

[71] Wong LL, Armugam A, Sepramaniam S (2015). Circulating microRNAs in heart failure with reduced and preserved left ventricular ejection fraction. Eur J Heart Fail.

[72] Zile MR, Desantis SM, Baicu CF (2011). Plasma biomarkers that reflect determinants of matrix composition identify the presence of left ventricular hypertrophy and diastolic heart failure. Circ Heart Fail.

[73] Kasner M, Gaub R, Westermann D (2011). Simultaneous estimation of NT-proBNP on top to mitral flow Doppler echocardiography as an accurate strategy to diagnose diastolic dysfunction in HFNEF. Int J Cardiol.

[74] Crowson CS, Myasoedova E, Davis JM (2011). Use of B-type natriuretic peptide as a screening tool for left ventricular diastolic dysfunction in rheumatoid arthritis patients without clinical cardiovascular disease. Arthritis Care Res.

[75] Nikolova AP, Hitzeman TC, Baum R (2018). Association of a novel diagnostic biomarker, the plasma cardiac bridging integrator 1 score, with heart failure with preserved ejection fraction and cardiovascular hospitalization. JAMA Cardiol.

[76] Bettencourt P, Ferreira A, Dias P (2000). Evaluation of brain natriuretic peptide in the diagnosis of heart failure. Cardiology.

[77] Karaca I, Gülcü E, Ferzeyn Yavuzkýr M (2007). B-type natriuretic peptide level in the diagnosis of asymptomatic diastolic dysfunction. Anatolian J Cardiol.

[78] Tv L, Fischer M, Hense HW (2005). BNP as a marker of diastolic dysfunction in the general population: importance of left ventricular hypertrophy. Eur J Heart Fail.

[79] Maisel AS, McCord J, Nowak RM (2003). Bedside B-Type natriuretic peptide in the emergency diagnosis of heart failure with reduced or preserved ejection fraction: results from the Breathing Not Properly Multinational Study. J Am Coll Cardiol.

[80] McCullough PA, Duc P, Omland T (2003). B-type natriuretic peptide and renal function in the diagnosis of heart failure: an analysis from the breathing not properly multinational study. Am J Kidney Dis.

[81] Buckley LF, Canada JM, Del Buono MG (2018). Low NT-proBNP levels in overweight and obese patients do not rule out a diagnosis of heart failure with preserved ejection fraction. ESC Heart Fail.

