# Supplementary material for: Natriuretic peptides for the detection of diastolic dysfunction and heart failure with preserved ejection fraction—a systematic review and meta-analysis
Source: BMC Med. 2020 Oct 30;18:290. doi: 10.1186/s12916-020-01764-x (PMC7599104; doi:10.1186/s12916-020-01764-x)
Supplement: Supplementary file 1 — Additional file 1. Search strategy for PubMed and Embase.com. [file 12916_2020_1764_MOESM1_ESM.docx]

**Additional File 1**

**Search string for PubMed and Embase.com**

**PubMed**

Heart failure

"Heart Failure"[Mesh:NoExp] OR heart failure*[tiab] OR cardiac failure*[tiab]

Normal ejection fraction

"Heart Failure, Diastolic"[Mesh] OR diastolic heart failure*[tiab] OR dhf[tiab] OR diastolic dysfunction*[tiab] OR diastolic heart dysfunction*[tiab] OR diastolic failure*[tiab] OR normal ejection fraction*[tiab] OR hfnef[tiab] OR preserved ejection fraction*[tiab] OR hfpef[tiab]

diagnostics

"Diagnosis"[Mesh] OR "Biomarkers"[Mesh:NoExp] OR "Biomarkers, Pharmacological"[Mesh] OR "Genetic Markers"[Mesh] OR "Echocardiography"[Mesh] OR "Electrocardiography"[Mesh] OR diagnos*[tiab] OR detection*[tiab] OR model[tiab] OR models[tiab] OR biomarker*[tiab] OR marker*[tiab] OR echocardiograph*[tiab] OR electrocardiograph*[tiab] OR doppler*[tiab] OR ECG[tiab]

Filter

NOT (Animals[Mesh] NOT Humans[Mesh]) NOT (mice[tiab] OR rats[tiab])

**Embase.com**

Heart failure

'heart failure'/de OR 'diastolic dysfunction'/exp OR 'heart left ventricle failure'/exp OR (heart NEAR/3 failure*):ab,ti OR (cardiac NEAR/3 failure*):ab,ti

Normal ejection fraction / linkerzijde

'diastolic heart failure'/exp OR (diastolic NEAR/3 failure*):ab,ti OR dhf:ab,ti OR (diastolic NEAR/3 dysfunction*):ab,ti OR ‘normal ejection fraction*’:ab,ti OR hfnef:ab,ti OR ‘preserved ejection fraction*’:ab,ti OR hfpef:ab,ti

Diagnostics

'diagnosis'/exp OR 'biological marker'/exp OR 'pharmacological biomarker'/exp OR 'genetic marker'/exp OR 'echocardiography'/exp OR 'electrocardiography'/exp OR diagnos*:ab,ti OR detection*:ab,ti OR model:ab,ti OR models:ab,ti OR biomarker*:ab,ti OR marker*:ab,ti OR echocardiograph*:ab,ti OR electrocardiograph*:ab,ti OR doppler*:ab,ti OR ECG:ab,ti

Filter
