# Supplementary material for: Natriuretic peptides for the detection of diastolic dysfunction and heart failure with preserved ejection fraction—a systematic review and meta-analysis
Source: BMC Med. 2020 Oct 30;18:290. doi: 10.1186/s12916-020-01764-x (PMC7599104; doi:10.1186/s12916-020-01764-x)
Supplement: Supplementary file 2 — Additional file 2: Additional information about the methods. Table S1. Information of the reference diagnoses of the 51 included studies. Table S2. Diagnostic performance measures of the 51 included studies. Table S3. Subgroup analyses stratified by geographic location or assay. [file 12916_2020_1764_MOESM2_ESM.docx]

**Additional File 2 – Supplementary methods**

*Data search*

Search terms included controlled terms (MesH in PubMed and Emtree in Embase), as well as free text terms. Duplicate articles were excluded.

*Study selection*

Studies were included if they: (i) studied a diagnostic performance measure (area under the receiver operating curve (AUC), sensitivity, specificity, positive predictive value (PPV), negative predictive value (NPV), positive likelihood ratio test (LR+), negative likelihood ratio test (LR-), accuracy, integrated discrimination improvement (IDI) or net reclassification improvement (NRI)), studied the performance of NPs for the detection of DD and/or HFpEF, (iii) included a control population without DD or HFpEF or with HFrEF, (iv) had a cross-sectional study design (maximum follow-up 2 years) and (v) were written in English or Dutch. We excluded studies if they: (i) studied the performance of the diagnostic marker for the detection of acute HF, (ii) in an ‘exotic’ patient population (e.g. Beta thalassemia, “Zebra” diagnosis heart failure-amyloid, hypertrophic cardiomyopathy or infiltrative disorders), (iii) used a single echo marker as reference standard, (iv) studied the performance of the diagnostic markers in a study design with a follow-up time of longer than two years, (v) were a RCT, (vi) were performed in animals or (vii) were a review, a systematic review, meta-analysis, editorial or conference abstract.

*Quality assessment*

A domain was rated as high risk of bias when one of the two or two of the three support questions were answered in a negative manner. However, for certain essential support questions such as the one about avoiding case-control design in domain ‘patient selection’ or about the right classification of the reference standard in domain ‘reference standard’, a negative answer would immediately lead to a high risk of bias for the respective domains. Study quality was assessed in terms of the reported diagnostic performance of the relevant marker and the detection of either DD or HFpEF, even if this was not the primary analysis presented in the study.

Additional File 2: Table S1. Information of the reference diagnoses of the 51 included studies.

| Author, Country[Ref.] | Reference diagnosis | | | | | |
| --- | --- | --- | --- | --- | --- | --- |
| Ansari, EU^42^ | **Grade III diastolic dysfunction:**   - Septal e’ <8 cm/s - Lateral e’ <10 cm/s - LAVI ≥ 34 mL/m^2^ - E/A ≥2 - DT <160 ms - Average E/e’ ≥13 - ARdur>Adur by ≥30 ms - ∆E/A ≥0.5 (Valsalva Maneuver) | | | | | |
| Bakowski, EU^19^ | **LV** **impaired** **relaxation:**   - <50 years:   - IVRT >100 ms   - DT >220 ms   - E/A <1 - ≥50 years:   - IVRT >105 ms   - DT >280 ms   - E/A <0.8 | **LV** **impaired** **compliance:**  Pseudonormal mitral flow   - <50 years:   - DT 160-220 ms   - IVRT 70-100 ms   - E/A 1.0-1.5   - S/D <0.5   - ARdur>Adur by >30 ms   - VmaxAR >0.35 m/s - ≥50 years:   - DT 160-280 ms   - IVRT 70-105 ms   - E/A 0.8-1.5   - S/D <0.5   - ARdur>Adur by >0.35 ms   - VmaxAR >0.35 m/s | | | **LV** **impaired** **compliance:**  Restrictive mitral flow   - DT <160 ms and at least 2 of the following parameters:   - IVRT <70 ms   - E/A >1.5   - S/D <0.5   - ARdur>Adur by >30 ms   - AR >0.35 m/s | |
| Barragan, EU^43^ | **Normal diastolic function:**   - E/A 1-2 - IVRT <100 ms - DT <220 ms - AR <35 ms - S/D >1 - e’ >8 cm/s - E/e’ <10 | | | | | |
| Barutçuoğlu, EU^44^ | **Delayed** **relaxation:**   - E/A <1 - IVRT >100 ms - DT >220 ms | | **Pseudonormal** **filling:**   - E/A 1-2 - IVRT 60-100 ms - DT 150-200 ms - e’ <8 cm/s | | | **Restrictive** **filling:**   - E/A >2 - IVRT <60 ms - DT <150 ms |
| Bettencourt, EU^73^ | **Restrictive** **filling:**   - E/A >2 - DT <150 ms - IVRT <70 ms | | | **Impaired** **relaxation:**  at least two of the following criteria:   - E/A <1 - DT >240 ms - IVRT >105 ms | | |
| Corteville, USA^45^ | **Pseudonormal** **filling:**   - E/A 0.75-1.5 - S <D | | | **Restrictive** **filling:**   - E/A ≥1.5 - S <D | | |
| Crowson, USA^76^ | **Pseudonormal** **filling:**   - E/A 0.75-1.5 - DT >140 ms - E/e’ ≥10 - ∆E/A ≥0.5 (Valsalva Maneuver) - S <D - ARdur>Adur by >30 ms | | | **Restrictive** **filling:**   - E/A >1.5 - DT <140 ms - ∆E/A ≥0.5 or ∆E/A <0.5 - E/e’ ≥10 - S <D - ARdur>Adur by >30 ms | | |
| Goto, AS^46^ | - LVEF ≥50% - τ ≥48 ms | | | | | |
| Grewal, CA+EU^47^ | **Pseudonormal** **filling:**   - E/A 0.75-1.5 - 2 of the following parameters:   - AR >0.35 cm/s   - ARdur>Adur by >30 ms   - E/A reversal   - S<D | | | **Restrictive** **filling:**   - E/A ≥1.5 | | |
| Karaca, EU^74^ | - LVEF >55% - E/A <1.0 (<55 years) or E/A <0.8 (>55 years) - DT >240 ms - IVRT <100 ms - FPV <45 ms - S>D | | | | | |
| Kasner, EU^72^ | - LVEDP ≥16 mmHg - τ ≥48 ms - dP/dt_min_ ≥1100 mmHg/s - PCWP >12 mmHg - PCWP at stress >20 mmHg | | | | | |
| Lukowicz, EU^75^ | < 30 years:   - E/A <1 - IVRT >92 ms - LAD >45 mm - left atrial maximal area >20 cm^2^ - current diuretic therapy   30-50 years:   - E/A <1 - IVRT >100 ms - LAD >45 mm - left atrial maximal area >20 cm^2^ - current diuretic therapy   >50 years   - E/A <0.5 - IVRT >105 ms - LAD >45 mm - left atrial maximal area >20 cm^2^ - current diuretic therapy | | | | | |
| Mak, NA^48^ | E/e’ >15 | | | | | |
| Mannacio, EU^49^ | **Moderate** **diastolic** **dysfunction:**   - E/A 0.8-1.0 - DT 150-200 ms - IVRT 60-95 ms - S<D - MVAdur<PVAdur - septal e’ <8.0 cm/s - average E/e’ 9-14 | | | **Severe** **diastolic** **dysfunction:**   - E/A >2 - DT <160 ms - IVRT <60 ms - S<D - MVAdur<PVAdur - average E/e’ >14 | | |
| Martos, EU^37^ | **Impaired** **relaxation:**  <55 years:   - E/A <1 - DT >250 ms - IVRT >90 ms   ≥55 years:   - E/A <0.8 - DT >240 ms - IVRT >90 ms | | **Pseudonormal** **filling:**   - E/A=1-1.5 - DT >240 ms - IVRT <90 ms - E/A reversal <1 by Valsalva maneuver | | | **Restrictive** **filling:**   - DT <160 ms - One of the following criteria:   - Left atrial size >5 cm   - E/A >1.5   - IVRT <70 ms |
| Mishra, USA^53^ | **Moderate** **diastolic** **dysfunction:**   - Septal e’ <8 cm/s - lateral e’ <10 cm/s - LAV ≥34 ml/m^2^ - E/A 0.8-1.5 - DT 160-200 ms - average E/e’ 9-12 - ARdur>Adur by >30 ms - ∆E/A ≥0.5 by Valsalva maneuver | | | **Severe** **diastolic** **dysfunction:**   - Septal e’ <8 cm/s - lateral e’ <10 cm/s - LAV ≥34 ml/m^2^ - E/A ≥2 - DT <160 - average E/e’ ≥13 - ARdur>Adur by >30 ms - ∆E/A ≥0.5 by Valsalva maneuver | | |
| Mocan, EU^54^ | **Abnormal relaxation:**   - E/A <0.8 - DT >200 ms | | **Pseudonormalization:**   - E/A 0.8-1.5 - ∆E/A ≥0.5 by Valsalva maneuver - LAVI >34 mL/m^2^ | | | **Restriction:**   - E/A ≥2 - LAVI >34 mL/m^2^ - IVRT ≤60 msec |
| Ravassa, EU^55^ | **Impaired** **relaxation:**   - Abnormally low age-specific E/A ratio (<2.5^th^ age-percentile) - E/e′ ≤8.5 | | **Mildly-to-moderately** **elevated** **LV** **filling** **pressures:**   - E/e′ >8.5 - E/A ratio 2.5^th^ and 97.5^th^ age-percentile - Ad < ARd + 10 - LAVI >29 mL/m^2^) | | | **Combined** **dysfunction:**   - Abnormally low age-specific E/A ratio (<2.5^th^ age-percentile) - E/e′ > .5 |
| Redfield, USA^21^ | **Moderate diastolic dysfunction:**   - E/A 0.75-1.5 - Two of the following:   - AR >35 cm/s   - ARdur>Adur by >30 ms   - E/A reversal   - S/D <1 | | | **Severe diastolic dysfunction:**   - E/A ≥1.5 - In participants with atrial fibrillation: DT is used | | |
| Tekten, EU^56^ | **Delayed** **relaxation:**   - E/A <1 - IVRT >100 ms - DT >220 ms | | **Pseudonormal** **filling:**   - E/A 1-2 - IVRT 60-100 ms - DT 150-200 ms - e’ <8 cm/s | | | **Restrictive** **filling:**   - E/A >2 - IVRT <60 ms - DT <150 ms |
| Tschöpe, EU^17^ | **Impaired** **relaxation:**   - E/A <1 - DT >220 ms - S>D - AR <35 cm/s | | **Pseudonormal** **filling:**   - E/A 1-2 - DT 150-220 ms - S<D - AR >35 cm/s | | | **Restrictive** **filling:**   - E/A > 2 - DT <150 ms - S < D - AR >35 cm/s |
| Wei, AS^50^ | **Abnormal left ventricular relaxation:**  One of the following criteria   - E peak deceleration time >240 ms - IVRT <90 ms - E/A <1 (<55 years old) - E/A <0.8 (>55 years old | | | | | |
| Zapata, EU^51^ | **Impaired** **relaxation:**   - E/A <0.8 - DT >200 ms - lateral e’ <10 cm/s - lateral E/e’ <8 | | **Pseudonormal** **filling:**   - E/A 0.8-2.0 - lateral e’ <10 cm/s - lateral E/e’ 9-11 | | | **Restrictive** **filling:**   - E/A ≥2 - lateral e’ <10 cm/s - lateral E/e’ >12 |
| Zuber, EU^52^ | Unclear which cutoff values for which echocardiographic parameters were used. | | | | | |
| Arques, EU^32^ | Expert opinion (two cardiologists and one chest physician) based on data collected at presentation and during hospital stay | | | | | |
| Arques, EU^31^ | - Chronic, isolated dyspnea - LVEF >50% - LV end-diastolic pressure >16 mmHg | | | | | |
| Baessler, EU^33^ | - Signs and symptoms of HF - LVEF >50% - e’ <10 cm/s & LA size >20 cm^2^ or LAD >48 mm - at least two of the following criteria:   - Lateral E/e’ >8   - E/A <0.8 & DT >200 ms or E/A >2 & DT <160 ms   - e’/a’ <0.9   - S <D   - ARdur-Adur >0 ms   - LVM >149 g/m^2^ (M) or LVM >122 g/m^2^ (F) | | | | | |
| Barroso, EU^34^ | - LVEF ≥50% - Grade II or III LVDD^29^ - With or without clinical symptoms or signs of HF   - Fluid retention (e.g. ankle swelling)   - Shortness of breath   - Reduced exercise intolerance   - Fatigue | | | | | |
| Berezin, EU^35^ | - Signs and symptoms of HF - LVEF ≥50% | | | | | |
| Borlaug, USA^36^ | PCWP ≥25 mmHg | | | | | |
| Celik, EU^57^ | - Signs and symptoms of HF - LVEF ≥50% - e’ <a’ | | | | | |
| Cui, AS^58^ | - Hospitalization with HF - Acute exacerbation of chronic stable HF - LVEF ≥50% | | | | | |
| Cui, AS^30^ | - Signs and symptoms of HF (NYHA II-IV) - LVEF >50% - E/A <1 - Hypertension, coronary artery disease, diabetes or other comorbidities | | | | | |
| Kim, AS^59^ | - Signs and symptoms of HF (based on Framingham criteria) - LVEF >50% - E/e’ >15 - NT-proBNP >220 pg/mL | | | | | |
| Liu, AS^60^ | - HF signs and symptoms according to Framingham criteria by a trained physician - LVEF ≥50% | | | | | |
| Martos, EU^37^ | - One hospitalization for proven Class IV HF (chest X-ray confirmation of signs of pulmonary congestion and received diuretics) - Continued signs and symptoms of HF (at least NYHA class II) - LVEF >45% - Doppler abnormalities of DD (see above) | | | | | |
| Nikolova, USA^77^ | - Past evidence of objective signs of fluid overload in the absence of noncardiac contributors, as determined by an assessment by an advanced HF specialist - Prior hospitalization for HF - Invasive hemodynamic data confirming presence of elevated cardiac filling pressures | | | | | |
| Mason, EU^61^ | ESC 2007 criteria without LVEDVI^39^ | | | | | |
| Polat, EU^64^ | - History of symptoms of HF (NYHA class II or III) - LVEF >50% - LVEDVI <97 ml/m^2^ - LVDD based on ESC 2007 criteria^39^ | | | | | |
| Reddy, USA^38^ | Elevated pulmonary capillary wedge pressure at rest (≥15 mmHg) or during exercise (≥25 mmHg) | | | | | |
| Sanders-van Wijk, EU^39^ | - Signs and symptoms of HF - LVEF ≥50% | | | | | |
| Santhanakrishnan, AS^65^ | - Signs and symptoms of HF - LVEF ≥50% | | | | | |
| Shuai, AS^66^ | - Signs and symptoms of HF (diagnosis of HF confirmed by two cardiologists) - LVEF ≥50% | | | | | |
| Sinning, EU^40^ | - Shortness of breath (NYHA class II-IV) - Medical treatment for HF - LVEF ≥50% - E/e’ ≥12 or; - E/e’ of 8-12, and E/A ≤0.5 | | | | | |
| Stahrenberg, EU^67^ | - LVEF >50% - Elevated filling pressures, based on ASE/EAE 2009 criteria | | | | | |
| Toma, CA^68^ | - Signs and symptoms of HF - LVEF ≥50% - expert opinion | | | | | |
| Wang, AS^41^ | - Exertional dyspnea (functional NYHA class II-III) - history of signs and symptoms of HF (Framingham criteria) - LVEF >50% | | | | | |
| Watson, EU^62^ | - One hospitalization for proven Class IV HF (confirmed by attending cardiologist) - Continued signs and symptoms of HF (at least NYHA class II) - LVEF ≥50% - Doppler abnormalities of DD | | | | | |
| Wong, AS^69^ | - Validated HF (presentation with primary diagnosis of HF or management of HF within 6 months of an episode of HF decompensation) - LVEF ≥50% | | | | | |
| Wong, AS^70^ | - Documented HF decompensation (within 6 months before study inclusion) - LVEF ≥50% | | | | | |
| Zile, USA^71^ | - Signs or symptoms of HF (Framingham criteria, Boston criteria, exercise testing or quality of life questionnaire) - LVEF ≥50% - LVEDVI <90 mL/m^2^ - Evidence of LVDD (invasively or noninvasively) | | | | | |
| Zordoky, CA^63^ | - HF adjudication by cardiologists - LVEF >50% | | | | | |

*Abbreviations: EU = Europe; AS = Asia, CA = Canada ; LV = left ventricular; IVRT = isovolumetric relaxation time; DT = deceleration time; E = mitral E-wave maximum velocity; A = mitral A-wave maximum velocity; S = venous S-wave velocity; D = venous D-wave velocity; ARdur = retrograde venous A-wave duration; Adur = mitral A-wave duration; VmaxAR = retrograde venous A-wave maximum velocity; e’ = peak velocity of early diastolic mitral annular motion; LVEF = left ventricular ejection fraction; τ = isovolumetric relaxation time; AR = retrograde venous A-wave velocity; FPV = left ventricular flow propagation velocity; LVEDP = left ventricular end diastolic pressure; dP/dt_min_ = minimal rate of LV pressure change; PCWP = papillary capillary wedge pressure; LAD = left atrial diameter; MVAdur = mitral valve A flow duration; PVAdur = atrial reversal velocity duration; LAV = left atrial volume; LAVI = left atrial volume index; LVM = left ventricular mass; HF = heart failure; NT-proBNP = N-terminal prohormone of brain natriuretic peptide; NYHA = New York Heart Association; DD = diastolic dysfunction; ESC = European Society of Cardiology; LVEDVI = left ventricular end diastolic volume index; LVDD = left ventricular diastolic dysfunction; ASE = American Society of Echocardiography; EAE = European Association of Echocardiography*

Additional File 2: Table S2. Diagnostic performance measures of the 51 included studies.

| Author, Country[Ref.] | Marker (assay) | Sensitivity | Specificity | PPV | NPV | Cut-off value | AUC |
| --- | --- | --- | --- | --- | --- | --- | --- |
| *Diastolic* *dysfunction* | | | | | | | |
| Bakowski, EU^19^ | ANP  BNP (RIA) | 85%  95% | 90%  100% |  |  | 220.7 pg/mL  74.7 pg/mL | - |
| Bettencourt, EU^73^ | BNP (RIA) |  |  |  |  |  | 0.89 (0.78;1.00)‡ |
| Crowson, USA^76^ | BNP (FEIA) | 25.6% (16.2;35.1%)‡ | 93.6% (92.4;94.9%)‡ | 18.4% (11.3;25.5%) | 95.7% (94.7;96.8%) | **F (**45/84 yrs): 64/167 pg/mL  **M** (45/84 yrs): 35/93 pg/mL |  |
| Goto, AS^46^ | BNP (RIA) | 74.7% (65.8;83.7%)*‡ | 60.9% (53.9;67.8%)*‡ | 47.9% (39.7;56.1%)*‡ | 83.3% (77.1;89.6%)*‡ | 22.4 pg/mL | 0.71 (0.64;0.78)‡ |
| Grewal, CA+EU^47^ | BNP (RIA)  NT-proBNP |  |  |  |  | 100 pg/mL  300 pg/mL  600 pg/mL | 0.72 (0.64;0.80)‡  0.67 (0.58;0.75)‡  0.74 (0.66;0.81) |
| Karaca, EU^74^ | BNP (FEIA) | 80% (65.7;94.3%)*‡ | 100% (91.1;100%)*‡ | 100% (92.5;100%)† | 76.9% (60.7;93.1%)† | 37 pg/mL | 0.97 (0.91;1.03)‡ |
| Lukowicz, EU^75^ | BNP (RIA) | 60.5% | 54.5% | 4.3% | 97.6% | 8.7 pg/mL | 0.63 (0.55;0.72)‡ |
| Mak, NA^48^ | Log(BNP) (FEIA) | 94% (68;102%)‡  88% (60;100%)  75% (45;95%)  50% (23;77%) | 68% (57;77%)‡  82% (72;89%)  89% (81;95%)  93% (85;97%) | 36% (22;52%)‡  48% (29;68%)  53% (33;79%)  57% (26;85%) | 98% (91;100%)‡  97%(90;99%)  95% (87%98%)  91% (82;95%) | 90 pg/mL  173 pg/mL  279 pg/mL  402 pg/mL | 0.89 (0.82;0.96)‡ |
| Mannacio, EU^49^ | BNP (FEIA) | 80% (59.3;93.1%)‡  91% (83.2;96.1%) | 91% (83.6;96.6%)‡  85% (57.2;97.8%) | 95.2% (86.1;100%)†‡  97.6 (94.4%;100%)† | 72.2% (51.5;92.9%)†‡  60% (38.5%;81.5%)† | 300 pg/mL  120 pg/mL | 0.83 (0.72;0.89)‡  0.86 (0.76;0.93) |
| Martos, EU^37^ | BNP (FEIA) |  |  |  |  |  | 0.69 (0.55;0.83)‡ |
| Redfield, USA^21^ | BNP (FEIA) | 75% (68;82%)*‡  **M**: 81%  **F**: 71% | 69% (67;71%)*‡  **M**: 64%  **F**: 74% | 15% (13;18%)†‡ | 97% (96;98%)†‡ | 36.4 pg/mL  6.7 pg/mL  7.1 pg/mL |  |
| Wei, AS^50^ | BNP (FEIA) | 79% (68;89%)*‡ | 92% (86;98%)*‡ | 89% (81;97%)†‡ | 84% (76;92%)†‡ | 40 pg/mL | 0.90 (0.84;0.97)‡ |
| Zapata, EU^51^ | BNP (FEIA)  NT-proBNP | 78% (66.5;89.5%)*‡  76% (64.2;87.8%)*‡ | 53% (36.5;69.1%)*‡  53% (36.5;69.1%)*‡ | 69.6% (57.6;81.7%)†‡  69.1% (56.9;81.3%)†‡ | 63.3% (46.1;80.6%)†‡  61.3% (44.1;78.4%)†‡ | 125 pg/mL  390 pg/mL | 0.74 (0.63;0.84)‡  0.68 (0.57;0.80)‡ |
| Zuber, EU^52^ | BNP (FEIA) | 64% (35.2;92.1%)*‡ | 64% (56.1;72.6%)*‡ | 13.2% (4.1;22.3%)†‡ | 95.4% (91;99.8%)†‡ | 100-500 pg/mL |  |
| Ansari, EU^42^ | Log(NT-proBNP) |  |  |  |  |  | 0.80 |
| Barragan, EU^43^ | NT-proBNP | 91% (83;99%)*‡  **≥55 yrs**: 90%  **<55 yrs**: 91%  **F**: 92%  **M**: 84% | 86% (75;98%)*‡  **≥55 yrs**: 83%  **<55 yrs**: 86%  **F**: 85%  **M**: 93% | 89% (80;98%)†‡ | 89% (79;99%)†‡ | 55 pg/mL  **≥55 yrs**: 58 pg/mL  **<55 yrs**: 55 pg/mL  **F**: 55 pg/mL  **M**: 63 pg/mL | 0.94  **≥55 yrs**: 0.90  **<55 yrs**: 0.90  **F**: 0.94  **M**:0.89 |
| Barutçuoğlu, EU^44^ | NT-proBNP | 86.9% (80.9;92.9%)*  82.8% (76.1;89.5%)*  79.5% (72.4;86.7%)*  76.2% (68.7;83.8%)*  74.6% (66.9;82.3%)*  73.8% (66;81.6%)*‡ | 62.4% (53.5;70.9%)*  71.8% (63.3;79.6%)*  76.1% (68.9;84.1%)*  79.5% (72.6;87%)*  83.8% (77.5;90.6%)*  83.8% (77.5;90.6%)*‡ | 70.4% (62.9;77.5%)*  74.8% (67.5;82.1%)*  77.4% (70.3;84.9%)*  79% (72.2;76.8%)*  80.5% (75.7;89.8%)*  82% (75.5;89.7%)*‡ | 81.5% (74.3;90.1%)*  80.2% (72.6;87.8%)*  77.8% (71;85.9%)*  77% (69.2;84.1%)*  75.8% (69.1;83.6%)*  75% (68.5;83.1%)*‡ | 45 pg/mL  50 pg/mL  55 pg/mL  60 pg/mL  65 pg/mL  70 pg/mL | 0.86 (0.82;0.91)‡ |
| Corteville, USA^45^ | NT-proBNP | 85% (76.6;92.6%)*  40% (28.9;50.6%)*‡  45% (33.8;55.9%)* | 41% (36.9;44.4%)*  53% (49.6;57.2%)*‡  87% (84.7;89.8%)* | 15% (11.3;17.8%)*  9.3% (6.2;12.4%)†‡  29.7% (21.4;37.9%)† | 95.7% (93.3;98.1%)†  88.1% (84.9;91.3%)†‡  93% (91;95%)† | <100 pg/mL  100-500 pg/mL  >500 pg/mL | 0.76 (0.69;0.82) ‡ |
| Kasner, EU^72^ | NT-proBNP | 70% (61.4;78.8%)*‡ | 91% (85.5;98.1%)*‡ | 92.6% (86.9;98.3%)† | 68.7% (59.6;77.8%)† | 125 pg/mL | 0.84 |
| Mishra, USA^53^ | NT-proBNP | 28% (22.7;33.1%)*‡  11% (7.3;14.6%)* | 76% (74.5;77.5%)*‡  91% (90;92.1%)* | 10% (7.9;12.1%)†‡  10.5% (7;14%)† | 91.7% (90.6;92.8%)†‡  91.4% (90.4;92.4%)† | 75th percentile  90th percentile | 0.51 |
| Mocan, EU^54^ | Log(NT-proBNP) (ELISA) | 94% (87;100%)*‡ | 60% (41;79%)†‡ | 82% (71;92%)†‡ | 78% (59;97%)†‡ | 95 pg/mL | 0.7 |
| Ravassa, EU^55^ | NT-proBNP (EIA) | 18.3% (12.7;23.8%)†‡ | 93.2% (91;95.3%)†‡ | 47.9% (36.3;59.5%)†‡ | 76.8% (73.6;80.1%)†‡ | 125 pg/mL |  |
| Tekten, EU^56^ | NT-proBNP | 83%  76% | 91%  96% |  |  | 62 pg/mL  120 pg/mL |  |
| Tschöpe, EU^17^ | NT-proBNP | 69% (58.1;80.1%)*‡  75% (64.7;85.3%)*  74% (63;84%)*  72% (61.4;82.7%)*  56% (44.1;67.7%)*  53% (41.1;64.8%)*  19% (9.8;28.5%)* | 92% (84.5;99.5%)†‡  86% (76.4;95.6%)*  88% (79;97%)*  90% (81.7;98.3%)*  92% (84.5;99.5%)*  92% (84.5;99.5%)*  100% (96.3;100%)* | 92.2% (84.8;99.5%)†‡  87.9% (79.6;96.3%)†  89.3% (81.2;97.4%)†  90.7% (83;98.5%)†  90.5% (81.6;99.4%)†  90% (80.7;99.3%)†  100% (86.7;100%)† | 8.7% (57.6;79.8%)†‡  71.7% (60.3;83.1%)†  71% (59.7;82.3%)†  70.3% (59.1;81.5%)†  60.5% (49.5;71.5%)†  59% (48.1;69.9%)†  47.6% (38.1;57.2%)† | 120 pg/mL  90 pg/mL  100 pg/mL  110 pg/mL  130 pg/mL  140 pg/mL  304 pg/mL | 0.83 (0.79;0.89)‡ |
| *Heart* *failure* *with* *preserved* *ejection* *fraction* | | | | | | | |
| Arques, EU^32^ | BNP (FEIA) | 95.5% (86.8;100%)*‡  86.4% (72;100%)* | 73.7% (53.9;93.5%)*‡  89.5% (75.7;100%)* | 80.8% (65.6;95.9%)†  90.5% (77.9;100%)† | 93.3% (80.7;100%)†  85% (69.4;100%)† | 200 pg/mL  253 pg/mL | 0.93 (0.80;0.98)‡ |
| Arques, EU^31^ | BNP (FEIA) | 33.3% (9.5;57.2%)*‡  67% (42.8;90.5%)* | 100% (84.5;100%)*‡  73% (46.4;99.1%)* | 100% (69.6;100%)†  76.9% (54;99.8%)† | 52.4% (31;73.7%)†  61.5% (35.1;88%)† | 100 pg/mL  31 pg/mL | 0.76 (0.55;0.90)‡ |
| Borlaug, USA^36^ | BNP (unknown) | 0% | 100% |  |  |  | <0.70 |
| Martos, EU^37^ | BNP (FEIA) |  |  |  |  |  | 0.82 (0.7;0.91)‡ |
| Mason, EU^61^ | BNP (FEIA)  NT-proBNP | 63% (50.6;75.7%)*‡  68% (56.4;80.5%)*‡ | 61% (55.6;66.5%)*‡  56% (50.6;61.7%)*‡ | 23.1% (16.5;29.7%)†  22.4% (16.2;28.6%)† | 90% (85.9;94%)†  90.6% (86.4;94.7%)† | 110 pg/mL  477 pg/mL | 0.64 (0.56;0.71)‡  0.64 (0.57;0.71)‡ |
| Watson, EU^62^ | Log(BNP) (FEIA) |  |  |  |  |  | 0.66 (0.58;0.75)‡ |
| Zordoky, CA^63^ | Log(BNP)  (FEIA)  Log(NT-proBNP) | 75% (57.7;92.3%)*‡  66.7% (47.8;85.5%)†  79.2% (62.9;95.4%)*‡  58.3% (38.6;78.1%)† | 76.3% (62.8;89.8%)*‡  65% (44.1;85.9%)†  78.9% (66;91.9%)*‡  60% (38.5;81.5%)† | 66.7% (48.9;84.5%)†  69.6% (50.8;88.4%)†  70.4% (53.2;87.6%)†  62.6% (43.5;83.7%)† | 78.4% (65.1;91.6%)†  61.9% (41.1;82.7%)†  85.7% (74.1;97.3%)†  54.6% (33.7;75.4%)† | Not reported | **HFpEF/no HF**: 0.82 (0.68;0.92)‡  **HFpEF/HFrEF**:0.73 (0.56;0.87)  **HFpEF/no HF**:  0.85 (0.73;0.95)‡  **HFpEF/HFrEF**:0.70 (0.53;0.84)‡ |
| Baessler, EU^33^ | NT-proBNP |  |  |  |  |  | 0.56 |
| Barroso, EU^34^ | Log(NT-proBNP) |  |  |  |  |  | 0.84 (0.77;0.91)‡ |
| Berezin, EU^35^ | NT-proBNP |  |  |  |  |  | 0.68 (0.61;0.74)‡ |
| Celik, EU^57^ | NT-proBNP | 45% (33.2;56.7%)*‡ | 96% (90.6;100%)*‡ | 93.9% (85.8;100%)† | 55.8% (44.3;66.3%)† | 125 pg/mL | 0.73 (0.64;0.80)‡ |
| Cui, AS^58^ | Log(NT-proBNP) | 61% (54;68%)*‡ | 80% (66;94%)*‡ | 81% (75;88%)† | 33% (22;44%)† | 295.9 pg/mL | 0.81 (0.66;0.82)‡ |
| Cui, AS^30^ | Log(NT-proBNP) (ELISA)  MR-proANP |  |  |  |  |  | 0.52 (0.43;0.61)‡  0.84 (0.75;0.91) |
| Kim, AS^59^ | NT-proBNP |  |  |  |  |  | 0.82 (0.78;0.86)‡ |
| Liu, AS^60^ | NT-proBNP (ELISA) | 94% (87.4;100%)*‡ | 74% (61.8;86.2%)*‡ | 78.3% (67.9;88.8%)† | 92.5% (84.3;100%)† | 424.3 pg/mL | 0.88 (0.82;0.95)‡ |
| Nikolova, USA^77^ | NT-proBNP |  |  |  |  |  | 0.93 (0.88;0.99)‡ |
| Polat, EU^64^ | NT-proBNP (ELISA) | 100% (95.8;100%)*‡ | 100% (95.2;100%)*‡ | 100% (95.8;100%)† | 100% (95.2;100%)† | 234 pg/mL | 0.98 |
| Reddy, USA^38^ | Log(NT-proBNP) | 60% (54;66%)*‡  59% (53;65%)*  46% (40;52%)* | 77% (70;84%)*‡  77% (70;84%)*  85% (79;91%)* | 82% (77;88%)†  82% (77;88%)†  84% (79;91%)† | 94% (90;98%)†  51% (44;57%)†  47% (41;52%)† | 272 pg/mL  275 pg/mL  450 pg/mL | 0.73  0.68  0.66 |
| Sanders-van Wijk, EU^39^ | Log(NT-proBNP) |  |  |  |  |  | 0.67 (0.62;0.72)‡ |
| Santhanakrishnan, AS^65^ | NT-proBNP | 82% (71.4;92.7%)*‡ | 94% (87.4;100%)*‡ | 93.2% (85.7;100%)† | 83.9% (74.3;93.6%)† | 247.6 pg/mL | 0.93 (0.89;0.98)‡ |
| Shuai, AS^66^ | NT-proBNP | 38% (23.6;51.9%)*‡ | 94% (88.1;100%)*‡ | 85% (69.4;100%)* | 64% (53.5;74.8%)* | 220 pg/mL |  |
| Sinning, EU^40^ | Log(NT-proBNP) |  |  |  |  |  | 0.74 (0.62;0.86)‡ |
| Stahrenberg, EU^67^ | NT-proBNP | 74% (64.8;83.4%)*  55% (44.7;65.9%)*‡ | 80% (74.1;85.5%)*  97% (94.3;99.3%)*‡ | 62.4% (52.9;71.8%)†  88.7% (97.2;80.2%)† | 87.2% (82.2;92.2%)†  82.7% (77.7;87.7%)† | 120 pg/mL  220 pg/mL | 0.86 (0.81;0.91)‡ |
| Toma, CA^68^ | NT-proBNP (RAMP) |  |  |  |  |  | 0.66 (0.51;0.81)‡  M: 0.64 (0.54;0.94)  F: 0.44 (0.20;0.68) |
| Wang, AS^41^ | NT-proBNP |  |  |  |  |  | 0.70 (0.58;0.79)‡ |
| Wong, AS^69^ | NT-proBNP |  |  |  |  |  | 0.89 (0.77;1.00)‡ |
| Wong, AS^70^ | NT-proBNP | **Cohort 1**: 79% (70;89%)*  **Cohort 2**: 80% (74;86%)* | **Cohort 1**: 44% (35;53%)*  **Cohort 2**: 48% (40;56%)* | **Cohort 1**: 46% (37;55%)*  **Cohort 2**: 66% (59;72%)* | **Cohort 1**: 79% (68;88%)*  **Cohort 2**: 66% (57;75%)* | 125 pg/mL | **Cohort 1**: 0.72 (0.64;0.79)‡  **Cohort 2**: 0.69 (0.63;0.75)‡ |
| Zile, USA^71^ | NT-proBNP (ChLIA) |  |  |  |  |  | 0.65  (p-value=0.002) |

** confidence intervals computed by hand. † estimate and confidence intervals computed by hand. Reported cut-off values were transformed into the clinical relevant scale (not log-transformed).* ‡ *estimates and their respective confidence intervals were meta-analysed.*

*Abbreviations: EU = Europe; AS = Asia, CA = Canad ;PPV =positive predictive value; NPV = negative predictive value; AUC = area under the receiver operator curve; yrs = years; ANP = atrial natriuretic peptide; BNP = brain natriuretic peptide; RIA = radioimmunoassay; AMB = ambulatory; F = female; AF = atrial fibrillation; DD = diastolic dysfunction; FEIA = fluorescence immunoassay; SD = systolic dysfunction; RA = rheumatoid arthritis; NT-proBNP = N-terminal prohormone of brain natriuretic peptide; CAD = coronary artery disease, AP = angina pectoris; HFpEF = heart failure with preserved ejection fraction; HTN = hypertension; ICU = intensive care unit; DOE = dyspnoeic on exertion; CHD = coronary heart disease; CKD = chronic kidney disease; Q = quartile; MetS = metabolic syndrome; HF = heart failure; HFrEF = heart failure with reduced ejection fraction; NCD = non-cardiac dyspnoea; Cath = catheterization; CHF = chronic heart failure; DHF = diastolic heart failure; ChLIA = chemiluminescence immunoassay*

Additional File 2: Table S3. Subgroup and sensitivity analyses stratified by geographic location or assay.

| Marker | Subgroup | AUC | Sensitivity | Specificity |
| --- | --- | --- | --- | --- |
| *Heart failure with preserved ejection fraction* | | | | |
| NT-proBNP | *Geographic location* | Test-statistic p-value: 0.5 | Test-statistic p-value: 0.9 | Test-statistic p-value: 0.7 |
|  | European | 0.77 (0.69;0.87; I^2^=89.3%) | 67% (44;91%; I^2^=96.9%) | 87% (67;100%; I^2^=99.3%) |
|  | Other | 0.82 (0.73;0.90; I^2^=93.1%) | 69% (54;85%; I^2^=95.1%) | 84% (76;92%; I^2^=78.2%) |
|  | *Assay variation* | Test-statistic p-value: 0.6 | Test-statistic p-value: 0.6 | Test-statistic p-value: 0.6 |
|  | Roche | 0.82 (0.76;0.88; I^2^=87.4%) | 61% (51;71%;I^2^=87.5%) | 84% (74;95%; I^2^=96.1%) |
|  | Other | 0.70 (0.35;1.0; I^2^=97.5%) | 98% (92;100%;I^2^=68.1%) | 88% (62;100%; I^2^=94.1%) |
|  | *Decade of publication* |  |  |  |
|  | 2000-2009 | N/A | N/A | N/A |
|  | 2010-2019 | 0.80 (0.74;0.87; I^2^=92.1%) | 69% (56;81%: I^2^=96.9%) | 85% (76;94%; I^2^=96.9%) |
| BNP | *Geographic location* |  |  |  |
|  | European | 0.79 (0.66;0.92; I^2^=84.1%) | 65% (31;100%,I^2^=95.6%) | 78% (55;100%; I^2^=95.3%) |
|  | Other | N/A | N/A | N/A |
|  | *Assay variation* |  |  |  |
|  | FEIA | 0.79 (0.69;0.90; I^2^=79.2%) | 68% (43;93%; I^2^=92.8%) | 78% (61;95%; I^2^=92.2%) |
|  | RIA | N/A | N/A | N/A |
|  | *Decade of publication* | Test-statistic p-value: 0.08 |  |  |
|  | 2000-2009 | 0.88 (0.77;0.0.99;I^2^=58.6%) | N/A | N/A |
|  | 2010-2019 | 0.73 (0.77;0.99; I^2^=67.1%) | 59% (36;81%; I^2^=79.7%) | 79% (56;100%; I^2^=95.7%) |
| *Diastolic dysfunction* | | | | |
| NT-proBNP | *Geographic location* | Test-statistic p-value: 0.2 | Test-statistic p-value: 0.05 | Test-statistic p-value: 0.1 |
|  | European | 0.80 (0.71;0.90; I^2^=85.8%) | 69% (51;86%; I^2^=97.2%) | 82% (71;93%; I^2^=94.2%) |
|  | Other | 0.72 (0.63;0.81; I^2^=63.2%) | 33% (21;45%; I^2^=73.8%) | 65% (42;87%; I^2^=99.2%) |
|  | *Assay variation* |  | Test-statistic p-value: 0.6 | Test-statistic p-value: 0.9 |
|  | Roche | 0.77 (0.69;0.84; I^2^=84.6%) | 64% (47;80%; I^2^=96.3%) | 77% (65;89%, I^2^=97.5%) |
|  | Other | N/A | 51% (0-100%; I^2^=99.6%) | 78% (46;100%; I^2^=91.4%) |
|  | *Decade of publication* | Test-statistic p-value: 0.8 | Test-statistic p-value: 0.07 | Test-statistic p-value: 0.9 |
|  | 2000-2009 | 0.76 (0.67;0.85; I^2^=82.1%) | 67% (38;96%: I^2^=96.1%) | 77% (52;100%; I^2^=97.1%) |
|  | 2010-2019 | 0.78 (0.60;0.95; I^2^=87.8%) | 58% (36;81%; I^2^=98.4%) | 78% (66;90%; I^2^=98.4%) |
| BNP | *Geographic location* | Test-statistic p-value: 0.9 | Test-statistic p-value: 0.6 | Test-statistic p-value: 0.9 |
|  | European | 0.80 (0.69;0.90; I^2^=87.5%) | 78% (71;86%; I^2^=0%) | 78% (56;99%; I^2^=97.0%) |
|  | Other | 0.81 (0.70;0.91; I^2^=87.9%) | 69% (47;92%; I^2^=100%) | 77% (64;90%; I^2^=99.0%) |
|  | *Assay variation* | Test-statistic p-value: 0.07 |  |  |
|  | FEIA | 0.85 (0.77;0.93; I^2^=82.6%) | 72% (57;87%; I^2^=91.6%) | 80% (68;91%; I^2^=98.8%) |
|  | RIA | 0.73 (0.63;0.83; I^2^=82.2%) | N/A | N/A |
|  | *Decade of publication* | Test-statistic p-value: 0.5 | Test-statistic p-value: 0.2 | Test-statistic p-value: 0.8 |
|  | 2000-2009 | 0.82 (0.72;0.91; I^2^=90.1%) | 78% (73;83%; I^2^=5.7%) | 79% (65;93%; I^2^=97.6%) |
|  | 2010-2019 | 0.76 (0.68;0.83; I^2^=56.4%) | 64% (38;90%; I^2^=95.4%) | 75% (55;95%; I^2^=97.9%) |

N/A: when subgroups consisted of less than 2 studies, subgroup and sensitivity analyses could not be performed and therefore no estimates are reported.
