# Supplementary material for: Natriuretic peptides for the detection of diastolic dysfunction and heart failure with preserved ejection fraction—a systematic review and meta-analysis
Source: BMC Med. 2020 Oct 30;18:290. doi: 10.1186/s12916-020-01764-x (PMC7599104; doi:10.1186/s12916-020-01764-x)
Supplement: Supplementary file 3 — Additional file 3: Figure S1. Prisma Flow diagram of the included studies. Figure S2. Risk of Bias score and applicability assessment of the 51 included studies. Figure S3. Funnel plots for summary estimates of NPs for the detection of DD and HFpEF. [file 12916_2020_1764_MOESM3_ESM.doc]

Additional File 3: Figure S1. Prisma Flow diagram of the included studies*.*


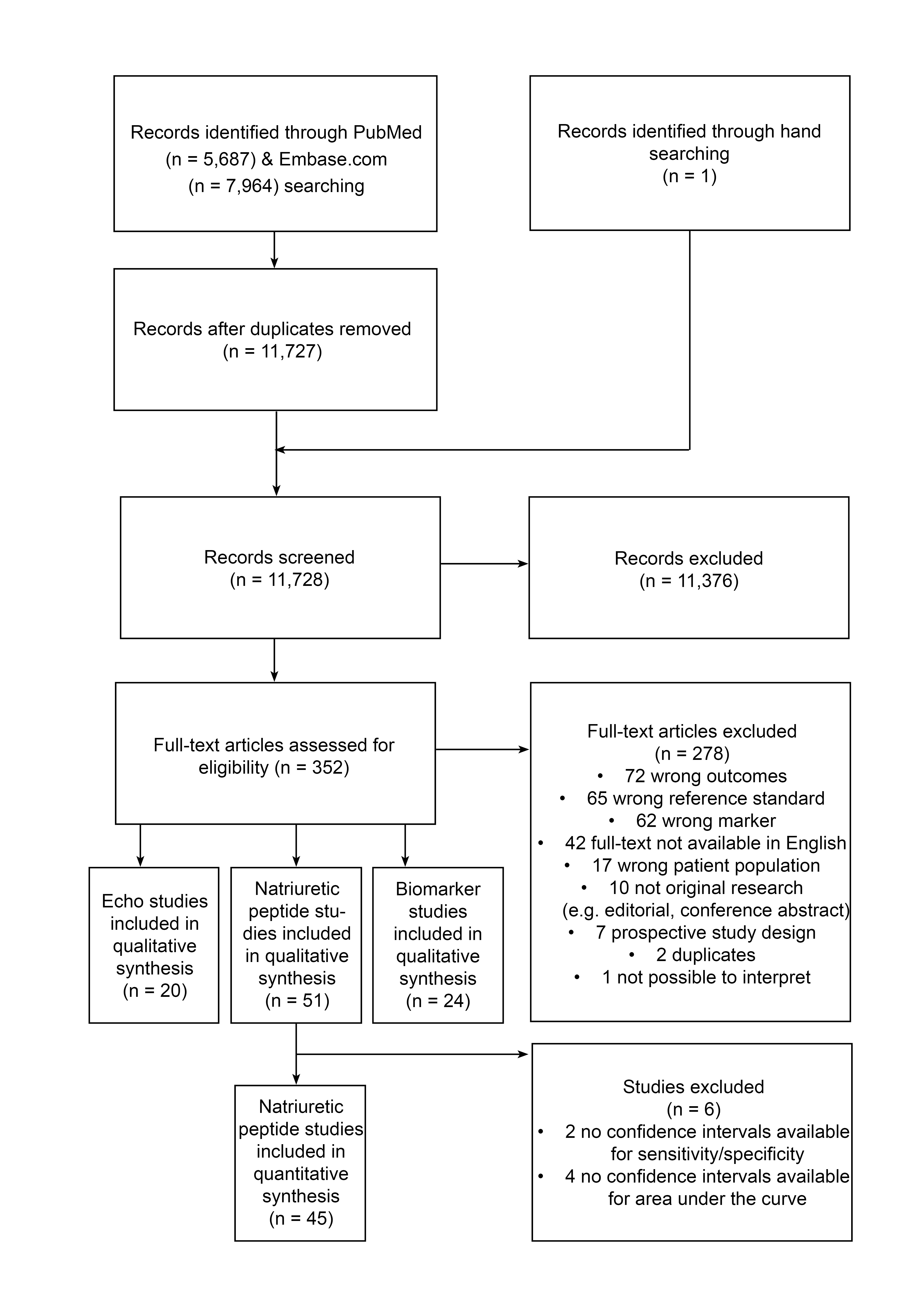


Additional File 3: Figure S2. Risk of Bias score and concerns regarding applicability assessment of the 51 included studies.


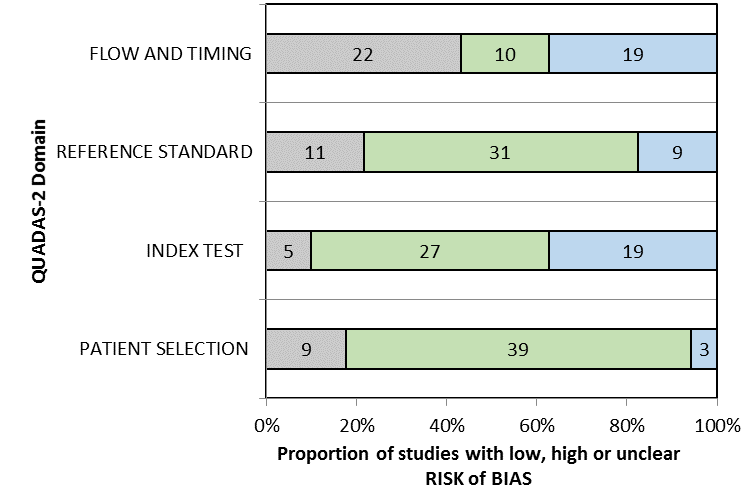

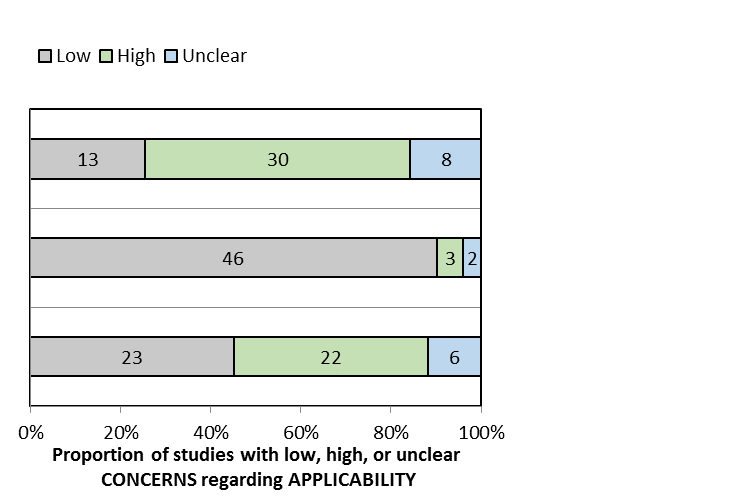

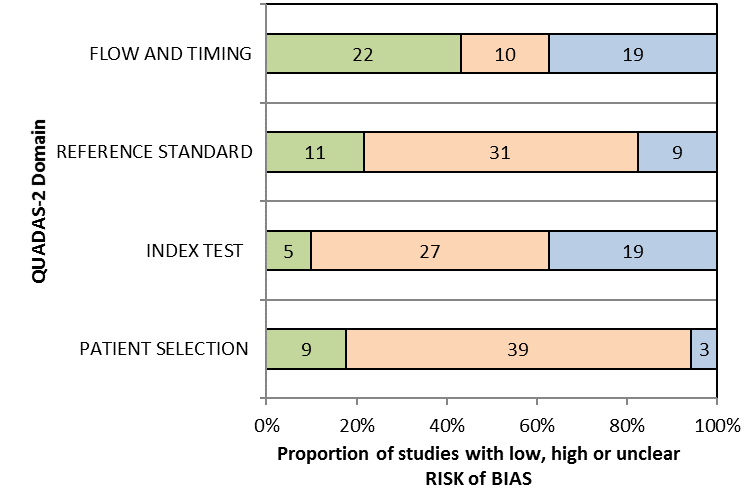

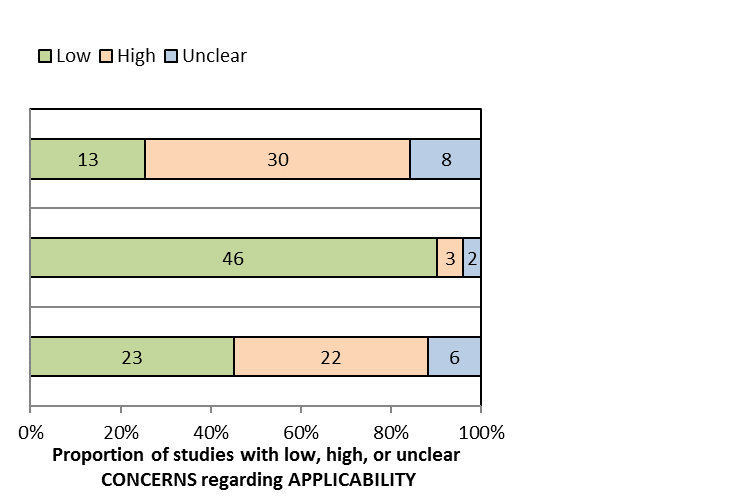


Additional File 3: Figure S3. Funnel plot for AUC values of NPs for the detection of DD and HFpEF and for sensitivity of NPs for the detection of DD and HFpEF. Black circles are the studies with NT-proBNP as diagnostic markers. Black triangles are the studies with BNP as diagnostic marker. Each data point indicates a study with the effect size (either AUC or sensitivity) on the x-axis and 1/standard error on the y-axis with the larger studies in the upper part. The dotted lines represent the 95%CI to visualize the symmetry around the pooled estimate. Symmetry would indicate no evidence of publication bias.


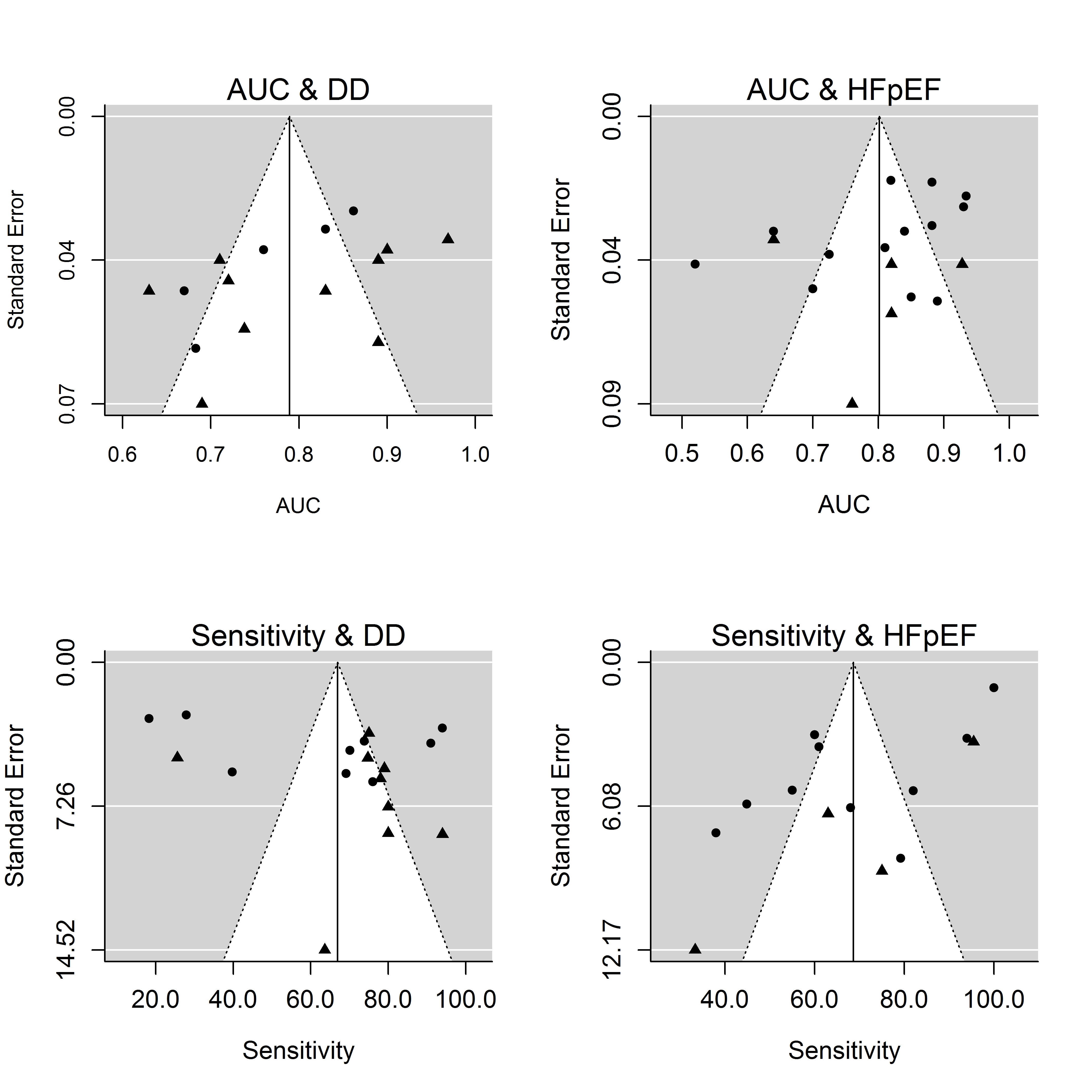


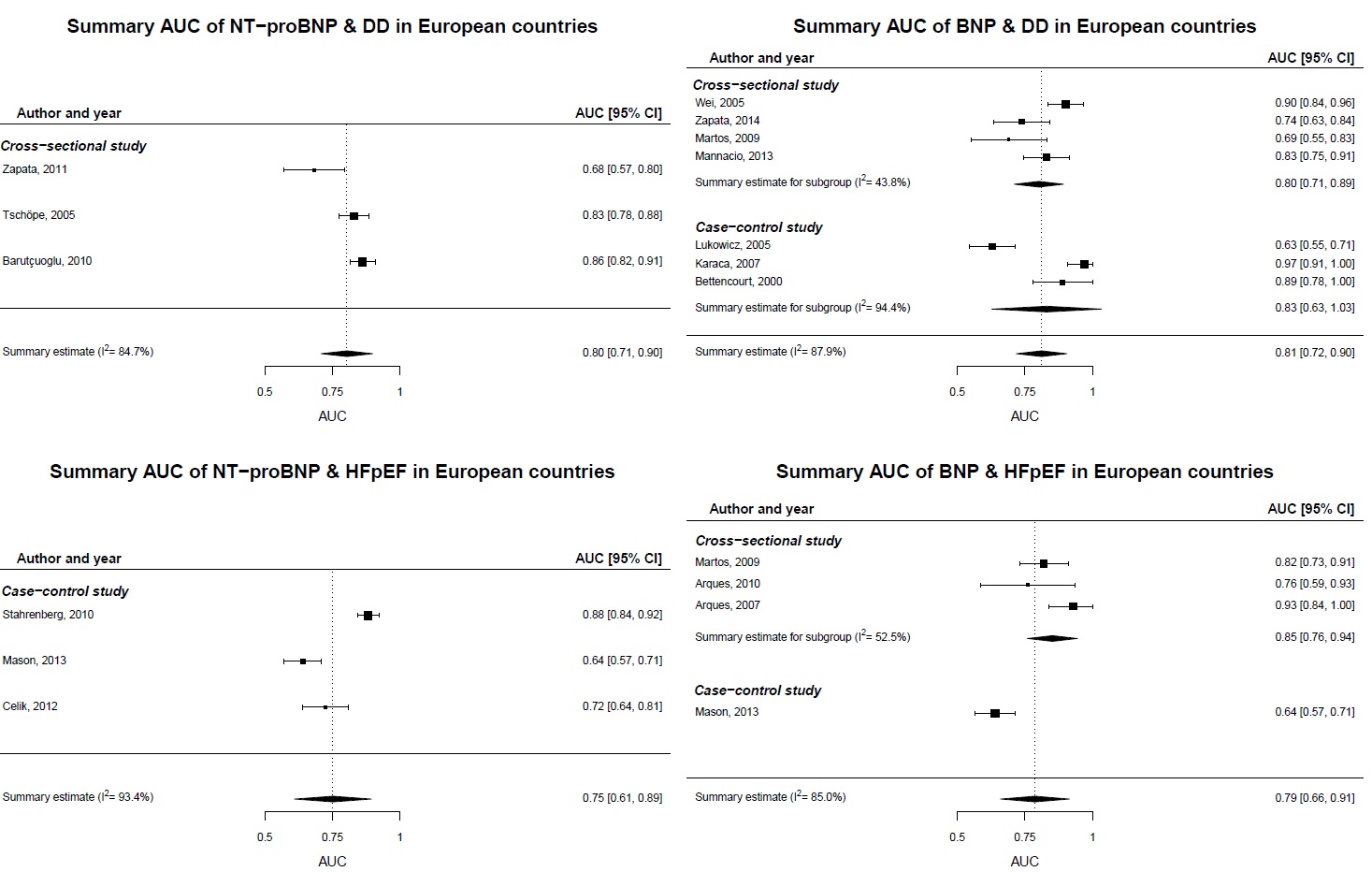


Supplementary figure 4. Meta-analysis of AUC values of NT-proBNP and BNP for the detection of DD with controls without DD, or for HFpEF with controls without HFpEF, in European countries.


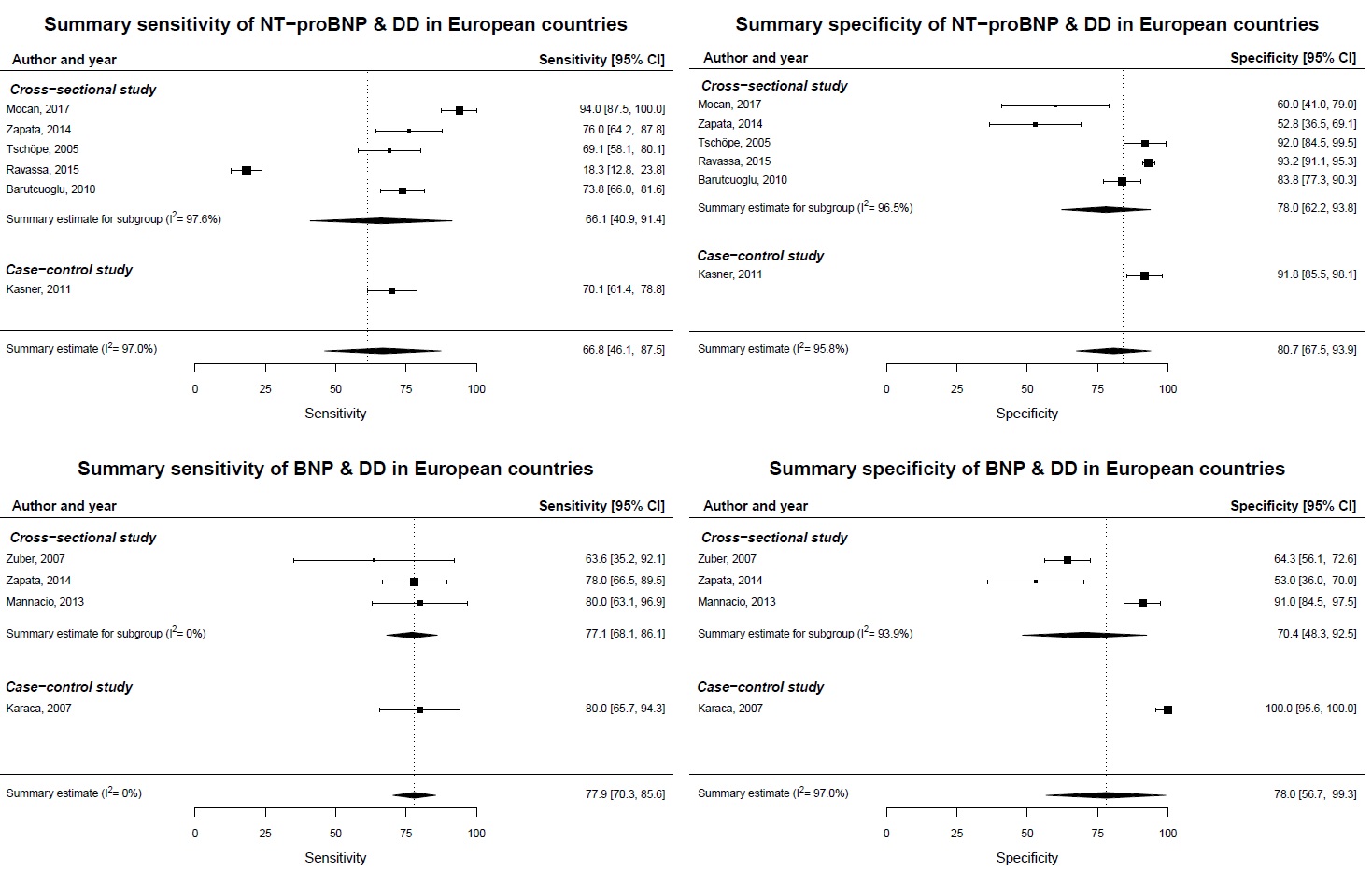


Supplementary figure 5. Meta-analysis of sensitivity and specificity of NT-proBNP and BNP for the detection of DD with controls without DD in European countries.


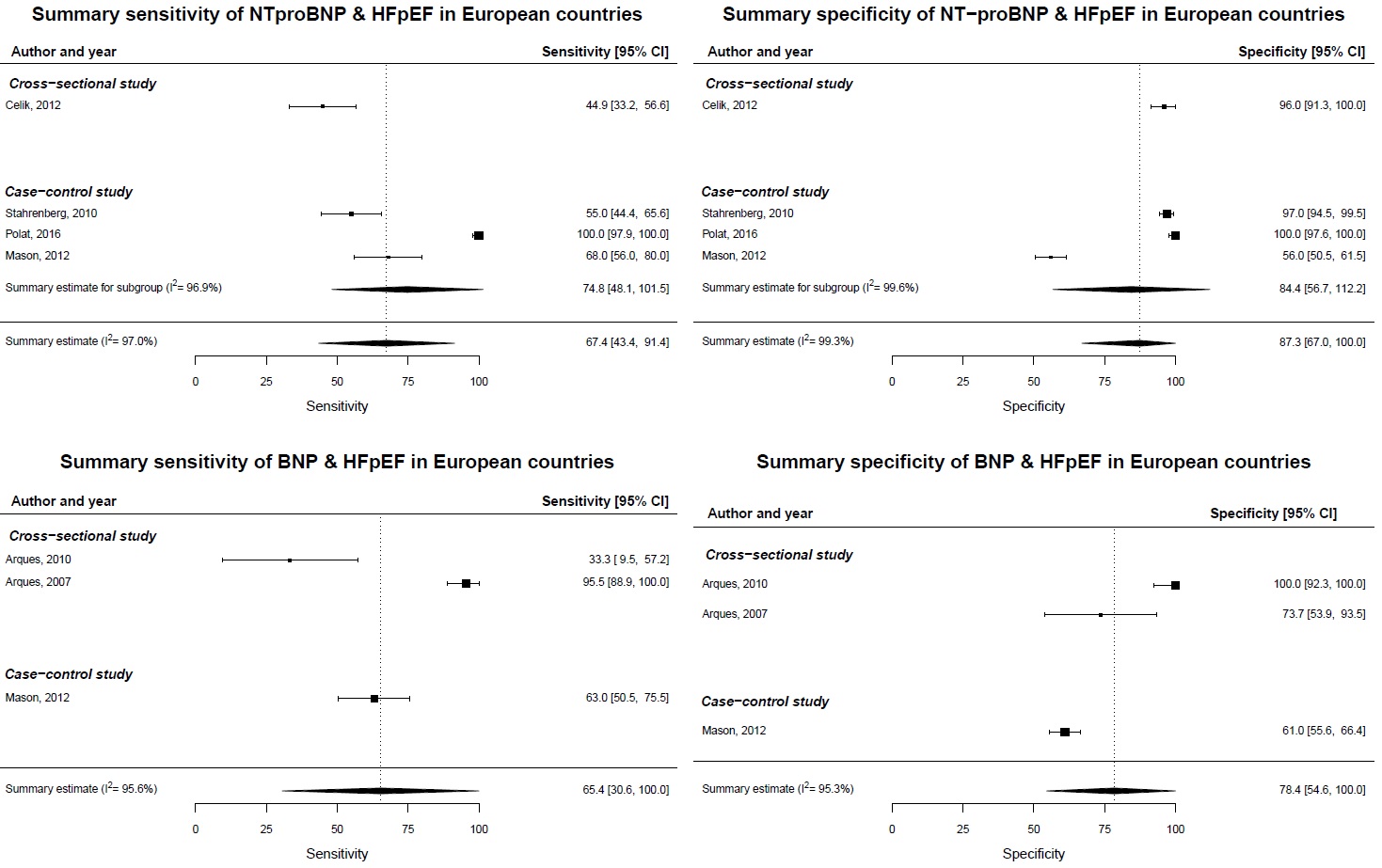


Supplementary figure 6. Meta-analysis of sensitivity and specificity of NT-proBNP and BNP for the detection of HFpEF with controls without HFpEF in European countries.
